# Base-Promoted Intramolecular Addition of Vinyl Cyclopropanecarboxamides to Access Conformationally Restricted Aza[3.1.0]bicycles

**DOI:** 10.3390/molecules28093691

**Published:** 2023-04-25

**Authors:** Jingya Li, Zhiguo Zhang, Liming Chen, Mengjuan Li, Xingjie Zhang, Guisheng Zhang

**Affiliations:** Key Laboratory of Green Chemical Media and Reactions, Ministry of Education, Henan Key Laboratory of Organic Functional Molecules and Drug Innovation, Collaborative Innovation Center of Henan Province for Green Manufacturing of Fine Chemicals, School of Chemistry and Chemical Engineering, Henan Normal University, Xinxiang 453007, China

**Keywords:** aza[3.1.0]bicycles, vinyl cyclopropanecarboxamides, amination, addition of alkenes

## Abstract

3-Azabicyclo[3.1.0]hexanes are common structural components in natural products and bioactive compounds. Traditionally, the metal-mediated cyclopropanation domino reaction of chain enzymes is the most commonly used strategy for the construction of this type of aza[3.1.0]bicycle derivative. In this study, a base-promoted intramolecular addition of alkenes used to deliver conformationally restricted highly substituted aza[3.1.0]bicycles is reported. This reaction was tailor-made for saturated aza[3.1.0] bicycle-containing fused bicyclic compounds that may be applied in the development of concise and divergent total syntheses of bioactive compounds.

## 1. Introduction

**Figure 1:**
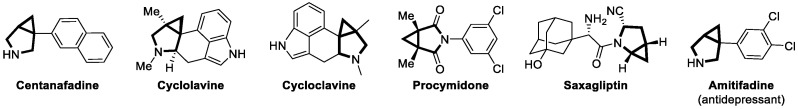
Bioactive molecules containing aza[3.1.0]bicycles.

Saturated *N*-heterocycles such as 3-azabicyclo[3.1.0]hexanes are common structural components in natural products and bioactive compounds with a broad spectrum of activity against various bacteria, mycobacteria, parasites, tumors, and neurological disorders (Figure 1) [1,2,3,4,5,6,7,8,9]. For example, Duocarmycin SA, Yatakemycin, and CC-1065 are representative members of such well-known biomolecules that derive their antitumor activity from their ability to alkylate DNA [10]. Furthermore, they have been identified as useful synthons in a range of organic transformations [11,12,13,14,15,16,17,18,19]. Consequently, the development of methods enabling the efficient construction of such structures has been a research focus in organic chemistry.

**Scheme 1:**
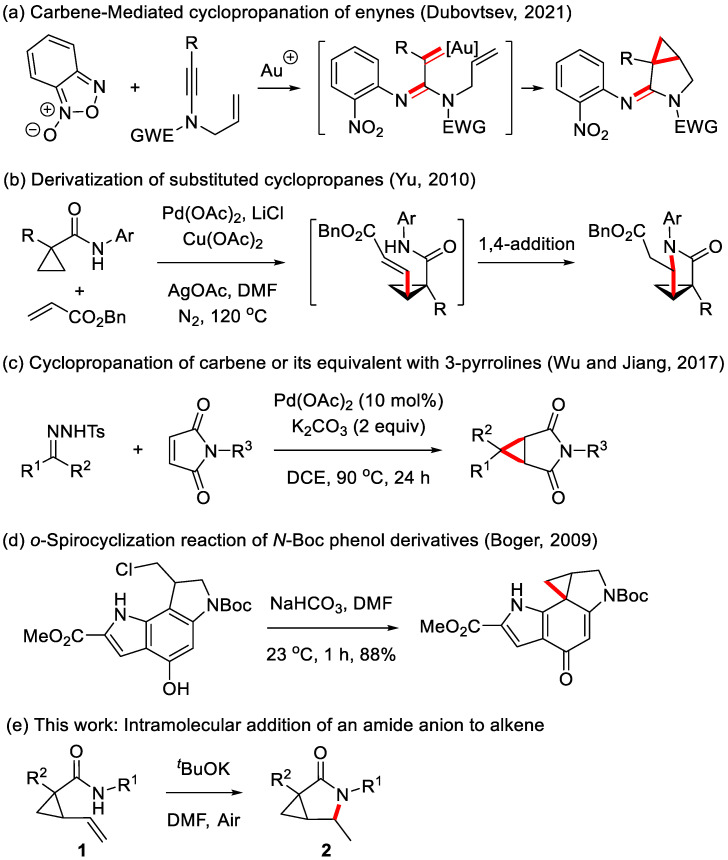
Strategies for constructing a bicyclic system [21,52,58,68].

A literature review indicates that the metal-mediated cyclopropanation domino reaction of chain enynes is the most commonly used strategy for the construction of aza[3.1.0]bicycle derivatives in terms of scalability and substrate scope, which highly rely on the in situ-generated metal carbene species in the presence of Pd, Au, Ru, Co, Ni, and Rh salts as catalysts [20,21,22,23,24,25,26,27,28,29,30,31,32,33]. Occasionally, the same conversion starting from enyne analogues has also been achieved by a photocatalytic pathway [34,35,36] as well as metal-free organocatalytic processes [37,38,39,40,41,42], mechanisms that are similar to the metal carbene processes (Scheme 1a). Another two effective approaches for the synthesis of 3-azabicyclo[3.1.0]hexanes involve the derivatization reactions of substituted cyclopropanes, such as C(sp^3^)–H bond activated alkenylation/amination tandem reactions and intramolecular aminolysis reactions (Scheme 1b) [43,44,45,46,47,48,49,50,51,52,53], and the reaction of functionalized maleimide derivatives with one carbon donor generated in situ derived from substituted diazomethanes, bromo(nitro)methane, substituted α-diazoacetates, and *N*-tosylhydrazones via an intermolecular [2+1] fused-annulation reaction (Scheme 1c) [54,55,56,57,58,59,60,61,62,63,64]. In particular, the base-induced intramolecular spirocyclization method of the alkylation subunit precursor appeared to be a more efficient proprietary reaction to access 3-azabicyclo[3.1.0]hexane scaffold-containing natural products via aryl metal or radical dearomatization/cyclization reactions (Scheme 1d) [65,66,67,68,69,70,71,72,73,74,75,76,77]. Although remarkable processing has been achieved in the last decades, achieving the synthesis of the structurally versatile aza[3.1.0]bicycles through readily available starting materials and simple and efficient chemical transformation remains a challenge. Here, describe our recent effort on the base-promoted intramolecular addition of vinyl cyclopropanecarboxamides **1** to access conformationally restricted aza[3.1.0]bicycles core **2** (Scheme 1e).

## 2. Results and Discussion

### 2.1. Reaction Optimization

Very recently, we developed a palladium(II)-catalyzed intramolecular oxidative aza-Wacker-type reaction to access a series of highly substituted aza[3.1.0]bicycles, starting from readily available compounds **1**. Combined with our other works related to the derivatization reactions of amides and previous reports, we envisioned that compound **1** may continue to generate highly substituted aza[3.1.0] bicycles **2** via a molecular olefin aza-addition reaction under appropriate bases (Scheme 1e). With this assumption in mind, the model reagent 1-(4-chlorophenyl)-*N*-(*p*-tolyl)-2-vinyl cyclopropane-1-carboxamide (**1a**) was selected to explore the feasibility of the designed transformation; some key results are listed in Table 1. After many attempts, we found that the desired product 1-(4-chlorophenyl)-4-methyl-3-(*p*-tolyl)-3-azabicyclo[3.1.0]hexan-2-one (**2a**) was isolated in 82% yield in the presence of 4.0 equiv. of *^t^*BuOK in DMF after 24 h, along with 11% of recovered **1a**, which could not be consumed by prolonging the reaction time (Table 1, entry 1). Notably, when we added 4.2 equiv. of 18-crown-6 ether to the reaction [48,78], starting material **1a** was completely consumed within 24 h (Table 1, entry 2). However, considering that it did not significantly affect the reaction time and the yield of product **2a**, as well as the economy of the transformation, it was not added in the later experiments. Moreover, reactions performed at a lower or higher loading of *^t^*BuOK failed to give a higher yield of fuse-heterocycle **2a** (Table 1, entries 3–5). Similarly, lower or higher temperatures did not help improve the reaction efficiency (Table 1, entries 6–9). The yield of the target product **2a** was not increased when the reactions were carried out in the presence of four other types of bases, namely, K_3_PO_4_, NaH, NaOH, and Cs_2_CO_3_ (Table 1, entries 10–13). Other solvents, including MeCN, dioxane, toluene, NMP, and DMSO, all provided diminished or no yields of the product (Table 1, entries 14–18).

### 2.2. Substrate Scope

**Scheme 2:**
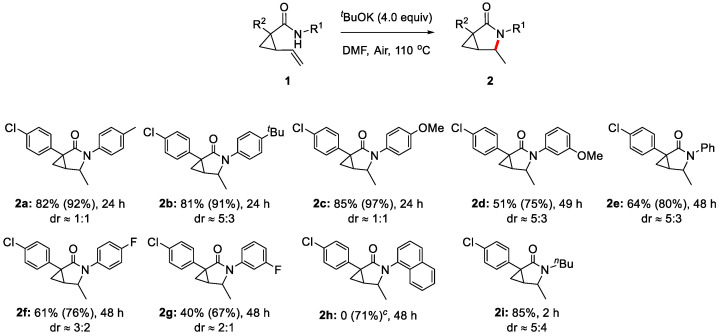
Extension of the reaction scope with various R^1^ ^*a*,*b*^. *^a^* Unless otherwise indicated, all these reactions were conducted with **1** (0.5 mmol, 1.0 equiv), *^t^*BuOK (4.0 equiv), and DMF (2 mL) at 110 °C under air in a sealed tube. *^b^* Isolated yields are reported, and unless otherwise indicated, the yields in parentheses are based on the conversion of substrate **1**. The dr values were determined from the ^1^H NMR analysis of the crude reaction mixture. *^c^* Recovery of **1h**.

With the identified optimal reaction conditions in hand, we evaluated the scope and drawbacks of this base-promoted intramolecular addition (Scheme 2). The variation in R^1^ was examined first. A variety of aryl groups having electron-releasing, -neutral, or -withdrawing groups at the 3- or 4-position of the benzene ring underwent smooth intramolecular annulation leading to the formation of the aza[3.1.0]bicycles **2a**–**g** in 40–85% yields with the regioselectivity ratio ranging from 1:1 to 2:1. Unfortunately, the analogous α-naphthyl-based substrate **1h** was not suitable for this system. *N*-Alkyl-substituted starting material **1i** afforded the desired product **2i** with excellent yield (85%) in *ca* 5:4 of dr value.

**Scheme 3:**
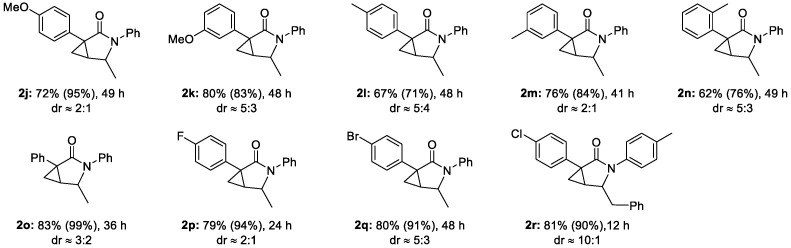
Extension of the reaction scope with various R^2^ ^*a*,*b*^. *^a^* Unless otherwise indicated, all these reactions were conducted with **1** (0.5 mmol, 1.0 equiv), *^t^*BuOK (4.0 equiv), and DMF (2 mL) at 110 °C under air in a sealed tube. *^b^* Isolated yields are reported, and the yields in parentheses are based on the conversion of substrate **1**. The dr values were determined from the ^1^H NMR analysis of the crude reaction mixture.

Next, the scope of the reaction was evaluated using different R^2^. Selected examples are presented in Scheme 3. It can be seen that the addition reaction was proved to be well tolerated by various 1-aryl-substituted vinyl cyclopropanecarboxamides bearing a MeO– (**2j** and **2k**), Me– (**2l**–**n**), F– (**2p**), and Br– (**2q**) group at the *para*-, *meta*-, or *ortho*-position, along with the phenyl group-substituted cyclopropane derivative (**2o**). Notably, the bromobenzene moiety of product **2q** retains a derivatization site for further functionalization reactions, including Suzuki–Miyaura [15,79,80,81,82], Buchwald–Hartwig [83,84,85], and Sonogashi coupling reactions [86,87,88,89,90]. In particular, the starting material **1r** with a styrene group on the cyclopropyl moiety provided the product **2r** with an 81% yield.

**Scheme 4:**
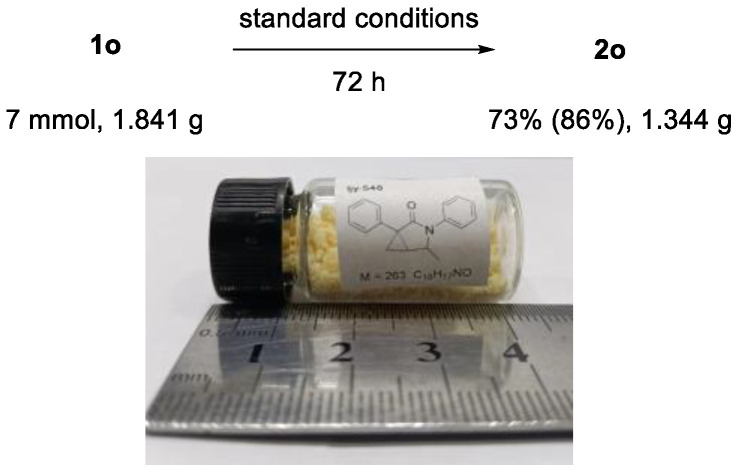
Gram-scale preparation.

With the aim of devising a practical, gram-scale synthesis of a biovaluable aza[3.1.0]bicycle scaffold, a reaction on 7 mmol (1.841 g) was carried out with this improved synthetic method based on the base-promoted intramolecular addition of alkenes. When we treated **1o** under optimal conditions, the reaction smoothly furnished a 73% yield of **2o** after 72 h under standard conditions, with 17% **1o** recovered (86% yield of **2o** based on the conversion of the substrate) (Scheme 4).

## 3. Materials and Methods

### 3.1. General Remarks

Unless stated otherwise, reactions were conducted in Schlenk under air. All reagents were purchased from commercial sources and used without further treatment unless otherwise indicated. Starting materials were synthesized following the literatures [91,92,93], and the procedures were described in the Supporting Information. DMF, CH_3_CN, DMSO, THF, and toluene for reactions were distilled under an atmosphere of dry N_2_. Petroleum ether (PE), used here, refers to the 60–90 °C boiling point fraction of petroleum. Ethyl acetate is abbreviated as EA. ^1^H NMR and ^13^C NMR spectra were recorded on a Bruker Avance/600 (^1^H: 600 MHz, ^13^C: 151 MHz) or Bruker Avance/400 (^1^H: 400 MHz, ^13^C: 101 MHz at 25 °C). Fluorine nuclear magnetic resonance (^19^F NMR) spectra were recorded on a Bruker Avance/600 spectrometer or a Bruker Avance/400. ^1^H NMR spectra were calibrated against residual CHCl_3_ in the solvent (7.26 ppm). ^13^C NMR spectra were calibrated against the peak of the residual CHCl_3_ in the solvent (77.2 ppm). NMR data are represented as follows: chemical shift (ppm), multiplicity (s = singlet, d = doublet, t = triplet, q = quartet, and m = multiplet), coupling constant in hertz (Hz), and integration. All high-resolution mass spectra (HRMS) were measured on a mass spectrometer by using electrospray ionization orthogonal acceleration time-of-flight (ESI-OA-TOF), and the purity of all samples used for HRMS (>95%) was confirmed by ^1^H NMR and ^13^C NMR spectroscopic analysis. All reactions were monitored by thin-layer chromatography (TLC) (PE: EA = 10:1) with GF254 silica gel-coated plates.

### 3.2. Typical Experimental Procedure for ***2*** (***2a*** as an Example)

In a Schlenk tube (25 mL), **1a** (156 mg, 0.5 mmol) and *^t^*BuOK (224 mg, 4.0 equiv.) were added. The mixture was stirred well in DMF (2 mL) and stirred at 110 °C in a sand bath under air (the whole process was closely monitored by TLC). After the completion of the reaction, DCM (5 mL) was added to water (10 mL) and extracted with dichloromethane (3 × 10 mL). Then the organic solvent was washed with H_2_O (15 mL) and saturated NaCl (15 mL) solutions, dried over anhydrous Na_2_SO_4,_ and concentrated in vacuo. The residue was purified by flash column chromatography with PE and EA (PE: EA = 20: 1) as eluent to give **2a** as a white solid (128 mg, 82%).

### 3.3. Characterization of Products

1-(4-Chlorophenyl)-4-methyl-3-(*p*-tolyl)-3-azabicyclo[3.1.0]hexan-2-one (**2a**). White solid. (Yield: 82%). Mp = 74–76 °C. dr ≈ 1:1. ^1^H NMR (600 MHz, CDCl_3_) *δ* 7.44–7.40 (m, 4H, Ar-H), 7.34–7.30 (m, 4H, Ar-H), 7.29 (dt, *J* = 9.0, 2.4 Hz, 2H, Ar-H), 7.17 (t, *J* = 7.2 Hz, 4H, Ar-H), 7.12 (d, *J* = 8.4 Hz, 2H, Ar-H), 4.53 (p, *J* = 6.0 Hz, 1H, N-CH), 4.20 (q, *J* = 6.4 Hz, 1H, N-CH), 2.38–2.35 (m, 2H, CH), 2.33 (s, 3H, Ar-CH_3_), 2.33 (s, 3H, Ar-CH_3_), 2.04 (dd, *J* = 7.8, 4.8 Hz, 1H, CH), 1.51 (dd, *J* = 7.8, 4.8 Hz, 1H, CH_2_), 1.39 (dd, *J* = 7.8, 4.8 Hz, 1H, CH_2_), 1.36 (d, *J* = 6.0 Hz, 3H, CH_3_), 1.31 (t, *J* = 4.5 Hz, 1H, CH_2_), 1.26 (t, *J* = 4.5 Hz, 1H, CH_2_), 1.19 (d, *J* = 6.0 Hz, 3H, CH_3_). ^13^C NMR (101 MHz, CDCl_3_) *δ* 173.5 (C=O), 172.4 (C=O), 135.8, 135.3, 135.1, 135.0, 134.9, 134.1, 133.1, 132.8, 130.1, 129.8, 129.61, 129.58, 128.6, 128.5, 124.6, 123.5, 56.4 (C-N), 53.1 (C-N), 34.4 (C), 33.4 (C), 26.9 (CH), 26.6 (CH), 21.5 (Ar-CH_3_), 21.0 (Ar-CH_3_), 20.9 (CH_3_), 20.1 (CH_3_), 16.9 (CH_2_), 16.8 (CH_2_). HRMS (ESI) (*m/z*) calculated for C_19_H_18_ClNO [M + Na]^+^: 334.0969, found: 334.0968. IR *v*/cm^−1^ (KBr) 1678, 1512, 1496, 1391, 1396, 1292, 1182, 1086, 1012, 839, 756, 718, 521.
3-(4-(*Tert*-butyl)phenyl)-1-(4-chlorophenyl)-4-methyl-3-azabicyclo[3.1.0]hexan-2-one (**2b**). White solid. (Yield: 81%). Mp = 119–121 °C. dr ≈ 5:3. ^1^H NMR (600 MHz, CDCl_3_) δ 7.42 (dd, *J* = 8.4, 3.6 Hz, 3.3H, Ar-H), 7.39 (s, 0.6H, Ar-H), 7.37 (s, 4.3H, Ar-H), 7.33 (d, *J* = 8.4 Hz, 2H, Ar-H), 7.29 (d, *J* = 8.4 Hz, 1.2H, Ar-H), 7.15 (d, *J* = 8.4 Hz, 1.2H, Ar-H), 4.55 (p, *J* = 6.0 Hz, 0.6H, minor, N-CH), 4.23 (q, *J* = 6.0 Hz, 1H, major, N-CH), 2.36 (dt, *J* = 7.8, 4.8 Hz, 0.6H, minor, CH), 2.04 (dd, *J* = 7.8, 4.2 Hz, 1H, major, CH), 1.51 (dd, *J* = 7.8, 4.8 Hz, 1H, major, CH_2_), 1.41 (dd, *J* = 7.8, 4.8 Hz, 0.8H, minor, CH_2_), 1.38 (d, *J* = 6.6 Hz, 3H, major, CH_3_), 1.32 (d, *J* = 4.8 Hz, 1H, minor, CH_2_), 1.31 (s, 14H, (CH_3_)_3_), 1.25 (t, *J* = 4.5 Hz, 1.3H, major, CH_2_), 1.21 (d, *J* = 6.0 Hz, 2H, minor, CH_3_). ^13^C NMR (151 MHz, CDCl_3_) δ 173.5 (minor, C=O), 172.4 (major, C=O), 148.8 (minor), 148.3 (major), 135.1 (major), 135.01 (major), 134.98 (minor), 134.0 (minor), 133.1 (major), 132.8 (minor), 130.1 (major), 129.7 (minor), 128.6 (major), 128.5 (minor), 125.9 (major), 125.8 (minor), 124.1 (minor), 122.9 (major), 56.2 (major, C-N), 53.0 (minor, C-N), 34.51 (minor, Ar-(CH_3_)_3_), 34.46 (major, Ar-(CH_3_)_3_), 34.4 (major, C), 33.4 (minor, C), 31.3 ((CH_3_)_3_), 27.1 (minor, CH), 26.5 (major, CH), 21.6 (major, CH_3_), 20.1 (minor, CH_3_), 17.0 (major, CH_2_), 16.8 (minor, CH_2_). HRMS (ESI) (*m/z*) calculated for C_22_H_24_ClNO [M + Na]^+^: 376.1439, found: 376.1431. IR *v*/cm^−1^ (KBr) 1675, 1515, 1493, 1374, 1291, 1266, 1188, 1103, 1067, 1011, 834, 796, 728, 550.
1-(4-Chlorophenyl)-3-(4-methoxyphenyl)-4-methyl-3-azabicyclo[3.1.0]hexan-2-one (**2c**). White solid. (Yield: 85%). Mp = 98–100 °C. dr ≈ 1:1. ^1^H NMR (400 MHz, CDCl_3_) *δ* 7.45–7.40 (m, 4H, Ar-H), 7.35–7.27 (m, 6H, Ar-H), 7.15–7.10 (m, 2H, Ar-H), 6.93–6.87 (m, 4H, Ar-H), 4.48 (p, *J* = 6.0 Hz, 1H, N-CH), 4.12 (q, *J* = 6.0 Hz, 1H, N-CH), 3.80 (s, 6H, Ar-OCH_3_), 2.39–2.32 (m, 1H, CH), 2.04 (dd, *J* = 7.6, 4.4 Hz, 1H, CH), 1.51 (dd, *J* = 7.6, 4.4 Hz, 1H, CH_2_), 1.38 (dd, *J* = 7.8, 5.0 Hz, 1H, CH_2_), 1.34 (d, *J* = 6.0 Hz, 3H, CH_3_), 1.30 (t, *J* = 4.8 Hz, 1H, CH_2_), 1.27 (t, *J* = 4.6 Hz, 1H, CH_2_), 1.17 (d, *J* = 6.4 Hz, 3H, CH_3_). ^13^C NMR (101 MHz, CDCl_3_) *δ* 173.5 (C=O), 172.4 (C=O), 157.7, 157.5, 135.04, 134.97, 133.1, 132.8, 130.4, 130.1, 129.7, 129.6, 128.6, 128.5, 126.4, 125.6, 114.3, 114.3, 56.8 (Ar-OCH_3_), 55.5 (C-N), 53.5 (Ar-OCH_3_), 34.2 (C), 33.4 (C), 26.8 (CH), 26.7 (CH), 21.5 (CH_3_), 20.2 (CH_3_), 17.0 (CH_2_), 16.9 (CH_2_). HRMS (ESI) (*m/z*) calculated for C_19_H_18_ClNO_2_ [M + Na]^+^: 350.0918, found: 350.0912. IR *v*/cm^−1^ (KBr) 1674, 1512, 1497, 1375, 1300, 1181, 1110, 1088, 1030, 832, 755, 716, 537.
1-(4-Chlorophenyl)-3-(3-methoxyphenyl)-4-methyl-3-azabicyclo[3.1.0]hexan-2-one (**2d**). White solid. (Yield: 51%). Mp = 78–80 °C. dr ≈ 5:3. ^1^H NMR (400 MHz, CDCl_3_) δ 7.44–7.39 (m, 3H, Ar-H), 7.35–7.27 (m, 3.7H, Ar-H), 7.26–7.22 (m, 1.8H, Ar-H), 6.96 (dd, *J* = 8.0, 1.2 Hz, 1H, major, Ar-H), 6.85 (t, *J* = 2.2 Hz, 0.6H, minor, Ar-H), 6.80 (dd, *J* = 8.0, 1.2 Hz, 0.6H, minor, Ar-H), 6.78–6.70 (m, 1.5H, Ar-H), 4.56 (p, *J* = 6.0 Hz, 0.6H, minor, N-CH), 4.27 (q, *J* = 6.4 Hz, 1H, major, N-CH), 3.80 (s, 4.5H, Ar-OCH_3_), 2.38 (dt, *J* = 7.9, 5.0 Hz, 0.6H, minor, CH), 2.06 (dd, *J* = 8.0, 4.4 Hz, 1H, major, CH), 1.51 (dd, *J* = 7.6, 4.8 Hz, 1H, major, CH_2_), 1.44–1.38 (m, 3.7H), 1.32 (t, *J* = 4.8 Hz, 1H, major, CH_2_), 1.26 (t, *J* = 4.4 Hz, 1.8H, CH_2_), 1.23 (d, *J* = 6.4 Hz, 2H, minor, CH_3_). ^13^C NMR (101 MHz, CDCl_3_) δ 173.5 (minor, C=O), 172.6 (major, C=O), 160.13 (major), 160.07 (minor), 139.2 (major), 138.0 (minor), 134.82 (minor), 134.81 (major), 133.2 (major), 132.9 (minor), 130.3 (major), 129.9 (minor), 129.60 (major), 129.55 (minor), 128.6 (major), 128.5 (minor), 116.5 (minor), 114.6 (major), 111.7 (minor), 111.1 (major), 110.6 (minor), 108.8 (major), 56.1 (minor, Ar-OCH_3_), 55.4 (major, C-N), 53.1 (major, Ar-OCH_3_), 34.8 (minor, C), 33.6 (major, C), 26.9 (minor, CH), 26.3 (major, CH), 21.5 (major, CH_3_), 20.0 (minor, CH_3_), 16.9 (major, CH_2_), 16.7 (minor, CH_2_). HRMS (ESI) (*m/z*) calculated for C_19_H_18_ClNO_2_ [M + Na]^+^: 350.0918, found: 350.0914. IR *v*/cm^−1^ (KBr) 1681,1602, 1579, 1490, 1456, 1373, 1293, 1173, 1087, 1068, 1037, 848, 756, 570.
1-(4-Chlorophenyl)-4-methyl-3-phenyl-3-azabicyclo[3.1.0]hexan-2-one (**2e**). White solid. (Yield: 64%). Mp = 88–90 °C. dr ≈ 5:3. ^1^H NMR (400 MHz, CDCl_3_) δ 7.49–7.45 (m, 2H, Ar-H), 7.43 (d, *J* = 8.4 Hz, 3H, Ar-H), 7.40–7.37 (m, 1.3H, Ar-H), 7.35 (d, *J* = 9.2 Hz, 2.8H, Ar-H), 7.33–7.28 (m, 2H, Ar-H), 7.25–7.21 (m, 1.3H, Ar-H), 7.18 (t, *J* = 7.4 Hz, 1.3H, Ar-H), 4.59 (p, *J* = 6.0 Hz, 0.6H, minor, N-CH), 4.27 (q, *J* = 6.4 Hz, 1H, major, N-CH), 2.42–2.35 (m, 0.6H, minor, CH), 2.06 (dd, *J* = 7.6, 4.4 Hz, 1H, major, CH), 1.52 (dd, *J* = 7.6, 4.8 Hz, 1H, major, CH_2_), 1.43–1.40 (m, 0.5H, minor, CH_2_), 1.38 (d, *J* = 6.0 Hz, 3H, major, CH_3_), 1.33 (t, *J* = 4.8 Hz, 0.7H, minor, CH_2_), 1.27 (t, *J* = 4.6 Hz, 1.5H, major, CH_2_), 1.21 (d, *J* = 6.0 Hz, 2H, minor, CH_3_). ^13^C NMR (151 MHz, CDCl_3_) δ 173.5 (minor, C=O), 172.5 (major, C=O), 137.8 (major), 136.8 (minor), 134.9 (major), 134.8 (minor), 133.2 (major), 132.9 (minor), 130.1 (major), 129.8 (minor), 129.0 (major), 128.9 (minor), 128.6 (major), 128.5 (minor), 125.9 (minor), 125.4 (minor), 124.5 (major), 123.2 (major), 56.1 (major, C-N), 53.0 (minor, C-N), 34.6 (major, C), 33.5 (minor, C), 26.9 (minor, CH), 26.5 (major, CH), 21.5 (major, CH_3_), 20.1 (minor, CH_3_), 16.9 (major, CH_2_), 16.8 (minor, CH_2_). HRMS (ESI) (*m/z*) calculated for C_18_H_16_ClNO [M + Na]^+^: 320.0813, found: 320.0807. IR *v*/cm^−1^ (KBr) 1680, 1595, 1491, 1374, 1294, 1178, 1102, 1065, 1039, 838, 753, 719, 528.
1-(4-Chlorophenyl)-3-(4-fluorophenyl)-4-methyl-3-azabicyclo[3.1.0]hexan-2-one (**2f**). White solid. (Yield: 61%). Mp = 82–84 °C. dr ≈ 3:2. ^1^H NMR (400 MHz, CDCl_3_) δ 7.44–7.36 (m, 5.3H, Ar-H), 7.35–7.31 (m, 2H, Ar-H), 7.31–7.28 (m, 2H, Ar-H), 7.22–7.16 (m, 1.3H, Ar-H), 7.11–7.02 (m, 3.2H, Ar-H), 4.52 (p, *J* = 6.0 Hz, 0.7H, minor, N-CH), 4.19 (q, *J* = 6.4 Hz, 1H, major, N-CH), 2.39 (dt, *J* = 8.0, 4.8 Hz, 0.7H, minor, CH), 2.07 (dd, *J* = 7.6, 4.4 Hz, 1H, major, CH), 1.53 (dd, *J* = 7.6, 4.8 Hz, 1H, major, CH_2_), 1.41 (dd, *J* = 7.6, 4.8 Hz, 1H, major, CH_2_), 1.36 (d, *J* = 6.0 Hz, 3H, major, CH_3_), 1.31 (t, *J* = 4.8 Hz, 0.8H, minor, CH_2_), 1.27 (t, *J* = 4.8 Hz, 2H, CH_2_), 1.20 (d, *J* = 6.4 Hz, 2H, minor, CH_3_). ^13^C NMR (151 MHz, CDCl_3_) δ 173.6 (minor, C=O), 172.5 (major, C=O), 160.5 (d, *J* = 246.1 Hz, minor, C-F), 160.2 (d, *J* = 244.6 Hz, major, C-F), 134.7 (major), 134.6 (minor), 133.6 (d, *J* = 3.0 Hz, major), 133.3 (major), 133.0 (minor), 132.7 (d, *J* = 2.7 Hz, minor), 130.1 (major), 129.8 (minor), 128.6 (major), 128.5 (minor), 126.4 (d, *J* = 4.5 Hz, minor), 125.4 (d, *J* = 7.6Hz, major), 115.9 (d, *J* = 4.5 Hz, minor), 115.8 (d, *J* = 6.0 Hz, major), 56.5 (major, N-CH), 53.3 (minor, N-CH), 34.3 (major, C), 33.4 (minor, C), 26.8 (minor, CH), 26.5 (major, CH), 21.4 (major, CH_3_), 20.1 (minor, CH_3_), 16.9 (major, CH_2_), 16.8 (minor, CH_2_). ^19^F NMR (565 MHz, CDCl_3_) δ −115.8 (minor), −116.4 (major). HRMS (ESI) (*m*/*z*) calculated for C_18_H_15_ClFNO [M + Na]^+^: 338.0718, found: 338.0716. IR *v*/cm^−1^ (KBr) 1682, 1505, 1378, 1180, 1101, 1064, 1014, 833, 718, 533.
1-(4-Chlorophenyl)-3-(3-fluorophenyl)-4-methyl-3-azabicyclo[3.1.0]hexan-2-one (**2g**). White solid. (Yield: 40%). Mp = 82–84 °C. dr ≈ 2:1. ^1^H NMR (400 MHz, CDCl_3_) δ 7.43–7.38 (m, 3.8H, Ar-H), 7.36–7.34 (m, 1.5H, Ar-H), 7.33–7.31 (m, 1.7H, Ar-H), 7.30 (d, *J* = 1.6 Hz, 0.5H, Ar-H), 7.29 (d, *J* = 3.6 Hz, 0.5H, Ar-H), 7.27–7.26 (m, 0.6H, Ar-H), 7.25–7.23 (m, 0.4H, Ar-H), 7.03 (dd, *J* = 8.8, 1.2 Hz, 1H, Ar-H), 6.93–6.83 (m, 1.4H, Ar-H), 4.57 (p, *J* = 6.0 Hz, 0.5H, minor, N-CH), 4.28 (q, *J* = 6.4 Hz, 1H, major, N-CH), 2.45–2.38 (m, 0.4H, minor, CH), 2.07 (dd, *J* = 8.0, 4.4 Hz, 1H, major, CH), 1.53 (dd, *J* = 7.6, 4.8 Hz, 1H, major, CH_2_), 1.45–1.39 (m, 3.5H), 1.32 (t, *J* = 4.6 Hz, 0.6H, minor, CH_2_), 1.28–1.22 (m, 3H). ^13^C NMR (151 MHz, CDCl_3_) δ 173.5 (minor, C=O), 172.6 (major, C=O), 163.0 (d, *J* = 246.1 Hz, major, C-F), 162.9 (d, *J* = 246.1 Hz, minor, C-F), 139.6 (d, *J* = 10.6 Hz, major), 138.4 (d, *J* = 10.6 Hz, minor), 134.5 (d, *J* = 3.0 Hz, major), 133.4 (major), 133.1 (minor), 130.2 (minor), 130.1 (d, *J* = 9.1 Hz, major), 130.0 (d, *J* = 9.1 Hz, minor), 129.9 (major), 128.7 (major), 128.5 (minor), 119.6 (d, *J* = 3.0 Hz, minor), 117.4 (d, *J* = 3.0 Hz, major), 112.6 (d, *J* = 6.0 Hz, minor), 111.8 (d, *J* = 21.1 Hz, major), 111.5, 109.8 (d, *J* = 25.7 Hz, major), 55.9 (major, N-CH), 52.9 (minor, N-CH), 34.8 (major, C), 33.6 (minor, C), 26.9 (minor, CH), 26.3 (major, CH), 21.3 (major, CH_3_), 20.0 (minor, CH_3_), 16.9 (major, CH_2_), 16.7 (minor, CH_2_). ^19^F NMR (565 MHz, CDCl_3_) δ -111.3 (major), -111.8 (minor). HRMS (ESI) (*m/z*) calculated for C_18_H_15_ClFNO [M + Na]^+^: 338.0718, found: 338.0712. IR *v*/cm^−1^ (KBr) 1682, 1588, 1491, 1452, 1368, 1296, 1185, 1087, 1064, 1014, 856, 754, 719, 591, 482.
3-Butyl-1-(4-chlorophenyl)-4-methyl-3-azabicyclo[3.1.0]hexan-2-one (**2i**). yellow oil. (Yield: 85%) °C. dr ≈ 5:4. ^1^H NMR (600 MHz, CDCl_3_) δ 7.35 (dd, *J* = 10.8, 8.4 Hz, 3H, Ar-H), 7.28 (d, *J* = 7.8 Hz, 3H, Ar-H), 3.98 (p, *J* = 6.0 Hz, 1H, minor, N-CH), 3.61–3.55 (m, 1.5H, minor), 3.54–3.48 (m, 1H, major, N-CH), 2.90–2.83 (m, 1.6H, major), 2.21–2.16 (m, 1H, major, CH), 1.86 (dd, *J* = 7.8, 4.2 Hz, 1H, minor, CH), 1.52–1.45 (m, 1.5H, major), 1.45–1.42 (m, 1H, minor), 1.41 (dd, *J* = 7.8, 4.8 Hz, 1.6H, major), 1.32 (d, *J* = 6.0 Hz, 2.7H, minor, CH_3_), 1.31–1.29 (m, 2H), 1.28–1.26 (m, 1.3H), 1.25 (d, *J* = 6.6 Hz, 3H, major, CH_3_), 1.08 (t, *J* = 4.8 Hz, 1H, major, CH_2_), 0.99 (t, *J* = 4.2 Hz, 0.8H, minor, CH_2_), 0.95–0.90 (m, 5H). ^13^C NMR (151 MHz, CDCl_3_) δ 173.9 (major, C=O), 173.2 (minor, C=O), 135.4 (minor), 135.3 (major), 132.8 (minor), 132.7 (major), 129.9 (minor), 129.7 (major), 128.5 (major), 128.4 (minor), 53.8 (minor, N-CH), 51.3 (major, N-CH), 39.8 (minor, N-CH_2_), 39.5 (major, N-CH_2_), 33.5 (minor, CH_2_-C-CH_2_), 33.2 (major, CH_2_-C-CH_2_), 29.9 (minor, CH), 29.5 (major, CH), 27.0 (minor, C), 26.9 (major, C), 21.0 (major, CH_2_-C-CH_3_), 20.2 (major, CH_2_-C-CH_3_), 20.1 (minor, CH_3_), 19.9 (minor, CH_3_), 17.1 (major, CH_2_), 16.6 (minor, CH_2_), 13.83 (minor, CH_2_-CH_3_), 13.79 (major, CH_2_-CH_3_). HRMS (ESI) (*m/z*) calculated for C_16_H_20_ClNO [M + Na]^+^: 300.1126, found: 300.1127. IR *v*/cm^−1^ (KBr) 1676, 1497, 1455, 1417, 1376, 1091, 1013, 819, 723, 527.
1-(4-Methoxyphenyl)-4-methyl-3-phenyl-3-azabicyclo[3.1.0]hexan-2-one (**2j**). White solid. (Yield: 72%). Mp = 48–50 ºC. dr ≈ 2:1. ^1^H NMR (400 MHz, CDCl_3_) δ 7.49 (d, *J* = 7.6 Hz, 2H, Ar-H), 7.42–7.32 (m, 6H, Ar-H), 7.25–7.13 (m, 2H, Ar-H), 6.93–6.84 (m, 3H, Ar-H), 4.59 (p, *J* = 6.0 Hz, 0.5H, minor, N-CH), 4.27 (q, *J* = 6.4 Hz, 1H, major, N-CH), 3.81 (s, 3H, major, Ar-OCH_3_), 3.80 (s, 1.4H, minor, Ar-OCH_3_), 2.34 (dt, *J* = 7.6, 4.8 Hz, 0.5H, minor, CH), 2.01 (dd, *J* = 7.6, 4.0 Hz, 1H, major, CH), 1.50 (dd, *J* = 7.6, 4.4 Hz, 1H, major, CH_2_), 1.41–1.36 (m, 4H), 1.29–1.24 (m, 1.5H, CH_2_), 1.23–1.19 (m, 2.8H). ^13^C NMR (151 MHz, CDCl_3_) δ 174.2 (minor, C=O), 173.3 (major, C=O), 158.9 (major), 158.7 (minor), 138.1 (major), 137.0 (minor), 130.2 (major), 129.9 (minor), 129.0 (major), 128.9 (minor), 128.4 (major), 128.3 (minor), 125.6 (minor), 125.1 (major), 124.5 (minor), 123.0 (minor), 114.0 (major), 113.8 (minor), 56.1 (major, Ar-OCH_3_), 55.4 (major, C-N), 55.3 (minor, Ar-OCH_3_), 53.0 (minor, C-N), 34.8 (major, C), 33.7 (minor, C), 26.6 (minor, CH), 26.2 (major, CH), 21.5 (major, CH_3_), 19.6 (major, CH_3_), 17.0 (minor, CH_2_), 16.2 (minor, CH_2_). HRMS (ESI) (*m/z*) calculated for C_19_H_19_NO_2_ [M + Na]^+^: 316.1308, found: 316.1308. IR *v*/cm^−1^ (KBr) 1682, 1516, 1492, 1392, 1369, 1296, 1177, 1107, 1031, 838, 761, 747, 538.
1-(3-Methoxyphenyl)-4-methyl-3-phenyl-3-azabicyclo[3.1.0]hexan-2-one (**2k**). White solid. (Yield: 80%). Mp = 78–80 °C. dr ≈ 5:3. ^1^H NMR (600 MHz, CDCl_3_) δ 7.48 (d, *J* = 7.8 Hz, 2H, Ar-H), 7.37 (dd, *J* = 16.6, 8.9 Hz, 3H, Ar-H), 7.28 (t, *J* = 7.8 Hz, 1H, Ar-H), 7.26–7.15 (m, 4H, Ar-H), 7.11 (s, 1H, Ar-H), 7.04 (d, *J* = 7.8 Hz, 1H, major, Ar-H), 6.99 (d, *J* = 7.8 Hz, 0.5H, minor, Ar-H), 6.84 (dd, *J* = 8.4, 2.4 Hz, 1H, major, Ar-H), 6.81 (dd, *J* = 7.8, 2.1 Hz, 0.5H, minor, Ar-H), 4.59 (p, *J* = 6.0 Hz, 0.6H, minor, N-CH), 4.26 (q, *J* = 6.0 Hz, 1H, major, N-CH), 3.83 (s, 3H, major, Ar-OCH_3_), 3.81 (s, 1.7H, minor, Ar-OCH_3_), 2.40 (dt, *J* = 7.8, 5.0 Hz, 0.6H, minor, CH), 2.06 (dd, *J* = 7.8, 4.2 Hz, 1H, major, CH), 1.55 (dd, *J* = 7.8, 4.8 Hz, 1H, major, CH_2_), 1.44 (dd, *J* = 7.8, 4.8 Hz, 0.6H, minor, CH_2_), 1.39 (d, *J* = 6.6 Hz, 3H, major, CH_3_), 1.32 (t, *J* = 4.8 Hz, 0.6H, minor, CH_2_), 1.25 (t, *J* = 4.5 Hz, 1H, major, CH_2_), 1.21 (d, *J* = 6.0 Hz, 1.7H, minor, CH_3_). ^13^C NMR (101 MHz, CDCl_3_) δ 173.7 (minor, C=O), 172.8 (major, C=O), 159.64 (major), 159.59 (minor), 138.0 (major), 137.9 (major), 136.9 (minor), 129.4 (major), 129.3 (minor), 129.0 (major), 128.9 (minor), 125.8 (minor), 125.3 (major), 124.6 (minor), 123.2 (major), 120.9 (major), 120.3 (minor), 114.7 (major), 114.0 (minor), 113.0 (minor), 112.8 (major), 56.1 (minor, Ar-OCH_3_), 55.3 (major, C-N), 53.0 (minor, Ar-OCH_3_), 35.1 (major, C), 33.9 (minor, C), 27.1 (minor, CH), 26.5 (major, CH), 21.4 (major, CH_3_), 20.0 (major, CH_3_), 17.0 (minor, CH_2_), 16.9 (major, CH_2_). HRMS (ESI) (*m/z*) calculated for C_19_H_19_NO_2_ [M + Na]^+^: 316.1308, found: 316.1308. IR *v*/cm^−1^ (KBr) 1682, 1594, 1493, 1456, 1372, 1293, 1186, 1040, 1028, 939, 759, 623.
4-Methyl-3-phenyl-1-(*p*-tolyl)-3-azabicyclo[3.1.0]hexan-2-one (**2l**). White solid. (Yield: 67%). Mp = 89–91 °C. dr ≈ 5:4. ^1^H NMR (600 MHz, CDCl_3_) δ 7.49 (d, *J* = 7.8 Hz, 2H, Ar-H), 7.39–7.34 (m, 7H, Ar-H), 7.25 (s, 0.7H, Ar-H), 7.20–7.13 (m, 5H, Ar-H), 4.59 (p, *J* = 6.0 Hz, 0.7H, minor, N-CH), 4.27 (q, *J* = 6.0 Hz, 1H, major, N-CH), 2.37–2.34 (m, 3.8H), 2.33 (s, 2H), 2.02 (dd, *J* = 7.8, 4.2 Hz, 1H, major, CH), 1.54 (dd, *J* = 7.8, 4.8 Hz, 1H, major, CH_2_), 1.42 (dd, *J* = 7.8, 4.8 Hz, 0.8H, minor, CH_2_), 1.39 (d, *J* = 6.0 Hz, 3H, major, CH_3_), 1.28 (t, *J* = 4.2 Hz, 1H, major, CH_2_), 1.23 (d, *J* = 4.2 Hz, 1H, major, CH_2_), 1.21 (d, *J* = 6.6 Hz, 2.4H, minor, CH_3_). ^13^C NMR (151 MHz, CDCl_3_) δ 174.1 (minor, C=O), 173.1 (major, C=O), 138.1 (major), 137.03 (major), 137.00 (minor), 136.7 (minor), 133.3 (major), 133.2 (minor), 129.2 (major), 129.1 (major), 128.95 (major), 128.85 (minor), 128.8 (minor), 128.5 (minor), 125.6 (minor), 125.1 (minor), 124.5 (major), 123.0 (major), 56.1 (major, C-N), 53.0 (minor), 35.0 (major, C), 33.9 (minor, C), 26.8 (minor, CH), 26.4 (major, CH), 21.5 (major, Ar-CH_3_), 21.15 (minor, Ar-CH_3_), 21.11 (minor, CH_3_), 19.6 (major, CH_3_), 17.0 (major, CH_2_), 16.2 (minor, CH_2_). HRMS (ESI) (*m/z*) calculated for C_19_H_19_NO [M + Na]^+^: 300.1359, found: 300.1358. IR *v*/cm^−1^ (KBr) 1678, 1595, 1493, 1456, 1371, 1296, 1179, 1110, 1066, 1031, 922, 762, 640, 529.
4-Methyl-3-phenyl-1-(*m*-tolyl)-3-azabicyclo[3.1.0]hexan-2-one (**2m**). White solid. (Yield: 76%). Mp = 103–105 °C. dr ≈ 2:1. ^1^H NMR (400 MHz, CDCl_3_) δ 7.52–7.48 (m, 2H, Ar-H), 7.40–7.30 (m, 5H, Ar-H), 7.26–7.14 (m, 5H, Ar-H), 7.13–7.05 (m, 1.6H, Ar-H), 4.59 (p, *J* = 6.0 Hz, 0.6H, minor, N-CH), 4.27 (q, *J* = 6.4 Hz, 1H, major, N-CH), 2.39–2.35 (m, 3.8H), 2.34 (s, 1.7H, minor), 2.04 (dd, *J* = 8.0, 4.4 Hz, 1H, major, CH), 1.57–1.54 (m, 1H, CH_2_), 1.45 (dd, *J* = 7.6, 4.8 Hz, 0.6H, CH_2_), 1.40 (d, *J* = 9.6 Hz, 3H, major, CH_3_), 1.29 (t, *J* = 4.8 Hz, 0.6H, minor, CH_2_), 1.24 (d, *J* = 4.4 Hz, 1H, CH_2_), 1.22 (d, *J* = 6.0 Hz, 2H, minor, CH_3_). ^13^C NMR (101 MHz, CDCl_3_) δ 174.0 (C=O), 173.1 (C=O), 138.1, 137.9, 137.0, 136.2, 136.1, 129.7, 129.5, 129.0, 128.9, 128.4, 128.3, 128.1, 127.8, 125.9, 125.7, 125.4, 125.2, 124.5, 123.1, 56.1 (C-N), 53.0 (C-N), 35.2 (C), 34.1 (C), 26.9 (CH), 26.4 (CH), 21.5 (Ar-CH_3_), 21.4 (CH_3_), 19.6 (CH_3_), 17.0, (CH_2_), 16.2 (CH_2_). HRMS (ESI) (*m/z*) calculated for C_19_H_19_NO_2_ [M + Na]^+^: 300.1359, found: 300.1359. IR *v*/cm^−1^ (KBr) 1683, 1494, 1455, 1376, 1296, 1181, 1108, 1067, 758, 606, 511.
4-Methyl-3-phenyl-1-(*o*-tolyl)-3-azabicyclo[3.1.0]hexan-2-one (**2n**). White solid. (Yield: 62%). Mp = 100–102 °C. dr ≈ 5:3. ^1^H NMR (600 MHz, CDCl_3_) δ 7.47 (d, *J* = 7.8 Hz, 2H, Ar-H), 7.40–7.31 (m, 4H, Ar-H), 7.27 (s, 0.5H, Ar-H), 7.25–7.14 (m, 7H, Ar-H), 4.66 (p, *J* = 6.0 Hz, 0.6H, minor, N-CH), 4.33 (q, *J* = 6.0 Hz, 1H, major, N-CH), 2.54 (s, 3H, major, Ar-CH_3_), 2.40 (s, 1.7H, minor, Ar-CH_3_), 2.22 (dt, *J* = 7.8, 4.8 Hz, 0.6H, minor, CH), 2.06 (dd, *J* = 7.2, 3.6 Hz, 1H, major, CH), 1.52 (dd, *J* = 7.8, 4.8 Hz, 1H, major, CH_2_), 1.47 (d, *J* = 4.8 Hz, 0.5H, minor, CH_2_), 1.45 (d, *J* = 6.0 Hz, 3H, major, CH_3_), 1.33–1.29 (m, 2H, CH_2_), 1.25 (d, *J* = 6.6 Hz, 2.4H, minor, CH_3_). ^13^C NMR (151 MHz, CDCl_3_) δ 173.9 (minor, C=O), 172.8 (major, C=O), 139.8 (major), 139.2 (minor), 138.0 (major), 137.1 (minor), 134.6 (minor), 134.3 (major), 130.7 (minor), 130.6 (major), 130.5 (major), 130.2 (minor), 128.95 (major), 128.89 (minor), 128.0 (major), 127.9 (minor), 125.9 (minor), 125.74 (major), 125.70 (minor), 125.2 (major), 124.5 (minor), 123.1 (major), 56.3 (major, C-N), 53.2 (minor, C-N), 35.5 (major, C), 34.7 (minor, C), 26.2 (minor, CH), 26.0 (major, CH), 20.9 (major, Ar-CH_3_), 20.1 (minor, Ar-CH_3_), 19.6 (minor, CH_3_), 19.5 (major, CH_3_), 17.1 (major, CH_2_), 14.6 (minor, CH_2_). HRMS (ESI) (*m/z*) calculated for C_19_H_19_NO_2_ [M + Na]^+^: 300.1359, found: 300.1358. IR *v*/cm^−1^ (KBr) 1682, 1596, 1495, 1456, 1373, 1293, 1179, 1099, 1067, 1027, 923, 751, 728, 659, 533.
4-Methyl-1,3-diphenyl-3-azabicyclo[3.1.0]hexan-2-one (**2o**). Yellow solid. (Yield: 83%). Mp = 87–89 °C. dr ≈ 3:2. ^1^H NMR (400 MHz, CDCl_3_) δ 7.52–7.46 (m, 5H, Ar-H), 7.41–7.33 (m, 6H, Ar-H), 7.32–7.28 (m, 1.2H, Ar-H), 7.28–7.26 (m, 1H, Ar-H),7.25–7.23 (m, 0.7H, Ar-H), 7.22–7.15 (m, 1.6H, Ar-H, 4.60 (p, *J* = 6.0 Hz, 0.7H, minor, N-CH), 4.28 (q, *J* = 6.3 Hz, 1H, major, N-CH), 2.43–2.37 (m, 0.7H, minor, CH), 2.07 (dd, *J* = 7.6, 4.4 Hz, 1H, major, CH), 1.58–1.55 (m, 1H, major, CH_2_), 1.46 (dd, *J* = 7.6, 4.8 Hz, 0.7H, minor, CH_2_), 1.39 (d, *J* = 6.0 Hz, 3H, major, CH_3_), 1.32 (t, *J* = 4.8 Hz, 0.7H, minor, CH_2_), 1.26 (t, *J* = 4.8 Hz, 1H, major, CH_2_), 1.22 (d, *J* = 6.0 Hz, 2H, minor, CH_3_). ^13^C NMR (151 MHz, CDCl_3_) δ 173.9 (minor, C=O), 173.0 (major, C=O), 138.0 (major), 136.9 (major), 136.3 (minor), 136.2 (minor), 129.0 (minor), 128.9 (major), 128.50, 128.47, 128.4, 127.3, 127.0, 125.7, 125.2, 124.6, 123.1, 56.1 (major, C-N), 53.0 (minor, C-N), 35.2 (major, C), 34.1 (minor, C), 26.9 (minor, C-H), 26.4 (major, C-H), 21.5 (major, CH_2_), 19.7 (minor, CH_2_), 17.0 (major, CH_3_), 16.4 (minor, CH_3_). HRMS (ESI) (*m/z*) calculated for C_18_H_17_NO [M + Na]^+^: 286.1202, found: 286.1202. IR *v*/cm^−1^ (KBr) 1682, 1598, 1495, 1447, 1372, 1299, 1178, 1101, 1063, 1021, 757, 664, 530.
1-(4-Fluorophenyl)-4-methyl-3-phenyl-3-azabicyclo[3.1.0]hexan-2-one (**2p**). White solid. (Yield: 79%). Mp = 98–100 °C. dr ≈ 2:1. ^1^H NMR (600 MHz, CDCl_3_) δ 7.48 (d, *J* = 7.8 Hz, 1.6H, Ar-H), 7.47–7.43 (m, 2.4H, Ar-H), 7.37 (q, *J* = 7.8 Hz, 2.5H, Ar-H), 7.24 (d, *J* = 7.8 Hz, 1H, Ar-H), 7.22–7.16 (m, 1.2H, Ar-H), 7.05 (t, *J* = 8.7 Hz, 1.6H, major, Ar-H), 7.02 (t, *J* = 8.7 Hz, 1H, minor, Ar-H), 4.59 (p, *J* = 6.0 Hz, 0.4H, minor, N-CH), 4.27 (q, *J* = 6.0 Hz, 1H, major, N-CH), 2.38 (dt, *J* = 7.8, 5.1 Hz, 0.4H, minor, CH), 2.05 (dd, *J* = 7.8, 4.2 Hz, 1H, major, N-CH), 1.51 (dd, *J* = 7.8, 4.2 Hz, 1H, major, CH_2_), 1.39 (d, *J* = 6.0 Hz, 3H, major, CH_3_), 1.31 (t, *J* = 4.5 Hz, 0.5H, minor, CH_2_), 1.26 (t, *J* = 4.5 Hz, 1H, major, CH_2_), 1.22 (d, *J* = 6.0 Hz, 1.3H, minor, CH_3_). ^13^C NMR (101 MHz, CDCl_3_) δ 173.8 (minor, C=O), 172.8 (major, C=O), 162.1 (d, *J* = 246.4 Hz, major, C-F), 162.0 (d, *J* = 246.4 Hz, minor, C-F), 137.9 (major), 136.8 (minor), 132.1 (d, *J* = 3.0 Hz, major), 132.0 (d, *J* = 4.0 Hz, minor), 130.6 (d, *J* = 8.1 Hz, major), 130.3 (d, *J* = 8.1 Hz, minor), 129.0 (major), 128.9 (minor), 125.8 (minor), 125.3 (major), 124.5 (minor), 123.2 (major), 115.3 (d, *J* = 22.2 Hz, major), 115.2 (d, *J* = 21.2 Hz, minor), 56.1 (major, C-N), 53.0 (minor, C-N), 34.7 (major, C), 33.6 (minor, C), 26.7 (minor, C-H), 26.3 (major, C-H), 21.5 (major, CH_2_), 19.9 (minor, CH_2_), 16.9 (major, CH_3_), 16.5 (minor, CH_3_). ^19^F NMR (376 MHz, CDCl_3_) δ -115.1 (major), -115.5 (minor). HRMS (ESI) (*m/z*) calculated for C_18_H_16_FNO [M + Na]^+^: 304.1108, found: 304.1107. IR *v*/cm^−1^ (KBr) 1685, 1493, 1455, 1406, 1394, 1381, 1250, 1230, 1076, 1066, 1028, 757, 600.
1-(4-Bromophenyl)-4-methyl-3-phenyl-3-azabicyclo[3.1.0]hexan-2-one (**2q**). White solid. (Yield: 80%). Mp = 119–121 °C. dr ≈ 5:3. ^1^H NMR (600 MHz, CDCl_3_) δ 7.49 (d, *J* = 8.4 Hz, 2H, Ar-H), 7.46 (t, *J* = 8.1 Hz, 3H, Ar-H), 7.40–7.34 (m, 6.5H, Ar-H), 7.24 (d, *J* = 7.8 Hz, 1H, Ar-H), 7.19 (dt, *J* = 11.4, 7.5 Hz, 1.6H, Ar-H), 4.58 (p, *J* = 6.0 Hz, 0.6H, minor, N-CH), 4.27 (q, *J* = 6.0 Hz, 1H, major, N-CH), 2.39 (dt, *J* = 7.8, 5.1 Hz, 0.6H, minor, CH), 2.06 (dd, *J* = 7.2, 4.8 Hz, 1H, major, CH), 1.52 (dd, *J* = 7.2, 4.8 Hz, 1H, major, CH_2_), 1.41 (dd, *J* = 7.8, 4.8 Hz, 1H, major, CH_2_), 1.38 (d, *J* = 6.6 Hz, 3H, major, CH_3_), 1.33 (t, *J* = 4.8 Hz, 0.7H, minor, CH_2_), 1.28 (t, *J* = 4.8 Hz, 1H, major, CH_2_), 1.21 (d, *J* = 6.6 Hz, 1.8H, minor, CH_3_). ^13^C NMR (101 MHz, CDCl_3_) δ 173.4 (minor, C=O), 172.4 (major, C=O), 137.8 (major), 136.7 (minor), 135.42 (major), 135.36 (minor), 131.6 (major), 131.4 (minor), 130.5 (major), 130.1 (minor), 129.0 (major), 128.9 (minor), 125.9 (minor), 125.4 (major), 124.6 (minor), 123.2 (major), 121.3 (major), 121.0 (minor), 56.1 (major, C-N), 53.0 (minor, C-N), 34.6 (major, C), 33.5 (minor, C), 26.9 (minor, CH), 26.5 (major, CH), 21.5 (major, CH_3_), 20.1 (minor, CH_3_), 16.9 (major, CH_2_), 16.8 (minor, CH_2_). HRMS (ESI) (*m/z*) calculated for C_18_H_16_BrNO [M + Na]^+^: 364.0307, found: 364.0307. IR *v*/cm^−1^ (KBr) 1682, 1489, 1389, 1382, 1100, 1066, 1057, 765, 699.
4-Benzyl-1-(4-chlorophenyl)-3-(*p*-tolyl)-3-azabicyclo[3.1.0]hexan-2-one: (**2r**). White solid. (Yield: 81%). Mp = 92–93 °C. dr ≈ 10:1. ^1^H NMR (600 MHz, CDCl_3_) δ 7.50 (d, *J* = 7.8 Hz, 2H, Ar-H), 7.43 (d, *J* = 7.8 Hz, 0.2H, Ar-H), 7.37 (d, *J* = 7.8 Hz, 0.5H, Ar-H), 7.36–7.27 (m, 3.5H, Ar-H), 7.24 (d, *J* = 7.8 Hz, 2.4H, Ar-H), 7.23–7.16 (m, 2.4H, Ar-H), 7.16–7.10 (m, 1.8H, Ar-H), 4.60–4.55 (m, 0.2H, N-CH), 4.49 (dd, *J* = 6.0, 3.4 Hz, 1H, N-CH), 3.01 (d, *J* = 13.8, 3.6 Hz, 1H, CH_2_), 2.94 (dd, *J* = 13.8, 6.6 Hz, 1H, CH_2_), 2.48–2.41 (m, 0.3H, CH_2_), 2.37 (s, 3H, major, CH_3_), 2.34 (s, 0.3H, minor, CH_3_), 2.31–2.45 (m, 0.2H, CH_2_), 2.17–2.12 (m, 0.2H, CH_2_), 2.06 (dd, *J* = 7.8, 4.8 Hz, 1H, CH_2_), 1.62–1.56 (m, 0.3H, CH_2_),1.52 (dd, *J* = 7.8, 5.4 Hz, 0.1H, CH_2_), 1.46 (dd, *J* = 7.7, 4.9 Hz, 1H), 1.27 (t, *J* = 4.4 Hz, 1H), 1.22–1.15 (m, 1H). ^13^C NMR (151 MHz, CDCl_3_) δ 173.5 (minor, C=O), 172.8 (major, C=O), 137.2, 136.1, 135.9, 135.2, 135.1, 134., 134.50, 134.1, 133.0, 132.9, 130.2, 129.9, 129.81, 129.78, 129.7, 129.1, 128.9, 128.7, 128.4, 128.3, 126.95, 126.88, 124.8, 122.9, 60.6 (major, N-CH), 59.2 (minor, N-CH), 39.2 (major, Ar-CH_2_), 37.5 (minor, Ar-CH_2_), 34.9 (major, CH), 33.8 (minor, CH), 24.9 (minor, C), 24.0 (major, C), 21.1 (minor, CH_3_), 21.0 (major, CH_3_), 19.0 (major, CH_2_), 17.3 (minor, CH_2_). HRMS (ESI) (*m/z*) calculated for C_25_H_22_ClNO [M + Na]^+^: 410.1282, found: 410.1275. IR *v*/cm^−1^ (KBr) 1681, 1514, 1494, 1384, 1293, 1082, 1066, 836, 749, 726, 531.

## 4. Conclusions

In summary, we developed a base-promoted intramolecular alkene addition reaction starting from readily available vinyl cyclopropanes to access a series of conformationally restricted biologically valuable highly substituted aza[3.1.0]bicycles in moderate to good yields. The transformation was performed in the presence of *^t^*BuOK in DMF at 110 °C under an air atmosphere. Experiments showed that large concentrations of the base are beneficial to the nucleophilic addition process. Although the protocol is limited to substituted cyclopropionamides with a range of functional aryl groups, the cyclopropane moiety in the fused ring is a valuable derivatization unit for the further construction of structurally diverse biologically organic molecules. This reaction was tailor-made for saturated aza[3.1.0]bicycle-containing fused bicyclic compounds. Further derivatization and chemical biology application evaluation of aza[3.1.0]bicyclic compounds are concurrently underway in our laboratory.

## Figures and Tables

**Table 1:** Survey of the reaction conditions *^a^.*

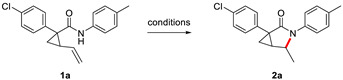					
Entry	Base/equiv	Solvent	t/°C	Time/h	Yield/% *^b^*
1	*^t^*BuOK (4.0)	DMF	110	24	82 (11)
2	*^t^*BuOK (4.0)	DMF	110	24	86 *^c^*
3	*^t^*BuOK (2.0)	DMF	110	24	64 (35)
4	*^t^*BuOK (3.0)	DMF	110	24	72 (6)
5	*^t^*BuOK (5.0)	DMF	110	24	57 (26)
6	*^t^*BuOK (4.0)	DMF	100	24	47 (48)
7	*^t^*BuOK (4.0)	DMF	120	24	77 (16)
8	*^t^*BuOK (4.0)	DMF	130	24	73 (8)
9	*^t^*BuOK (4.0)	DMF	140	24	43
10	K_3_PO_4_ (4.0)	DMF	110	24	39 (58)
11	NaH (4.0)	DMF	110	24	15 (81)
12	NaOH (4.0)	DMF	110	24	22 (68)
13	Cs_2_CO_3_ (4.0)	DMF	110	24	20 (72)
14	*^t^*BuOK (4.0)	MeCN	110	24	18 (76)
15	*^t^*BuOK (4.0)	Dioxane	110	24	0 (93)
16	*^t^*BuOK (4.0)	Toluene	110	24	0 (91)
17	*^t^*BuOK (4.0)	NMP	110	24	68 (6)
18	*^t^*BuOK (4.0)	DMSO	110	24	0 (85)

*^a^* Unless otherwise indicated, the reaction was conducted with **1a** (0.5 mmol, 1.0 equiv), base (4.0 equiv), and solvent (2 mL) at 110 °C under air in a sealed tube, and isolated yields are reported. *^b^* The recovery of **1a** is shown in parentheses. *^c^* In the presence of *^t^*BuOK (4.0 equiv) and 18-crown-6 ether (4.2 equiv).

## Data Availability

Not applicable.

## Supplementary Materials

The following supporting information can be downloaded at: https://www.mdpi.com/article/10.3390/molecules28093691/s1. Characterization data for product **2**, including ^1^H-NMR and ^13^C-NMR spectroscopies, are available online. References [91,92,93] are cited in the Supplementary Materials.

## Sample Availability

Samples of compounds are available from the authors.

## References

[1] Devarahosahalli Veeranna K., Kanti Das K., Baskaran S. (2021). Reversal of polarity by catalytic SET oxidation: Synthesis of azabicyclo[*m.n*.0]alkanes via chemoselective reduction of amidines. Org. Biomol. Chem..

[2] Lorthiois E., Anderson K., Vulpetti A., Rogel O., Cumin F., Ostermann N., Steinbacher S., Mac Sweeney A., Delgado O., Liao S.-M. (2017). Discovery of Highly Potent and Selective Small-Molecule Reversible Factor D Inhibitors Demonstrating Alternative Complement Pathway Inhibition In Vivo. J. Med. Chem..

[3] Kuttruff C.A., Ferrara M., Bretschneider T., Hoerer S., Handschuh S., Nosse B., Romig H., Nicklin P., Roth G.J. (2017). Discovery of BI-2545: A Novel Autotaxin Inhibitor That Significantly Reduces LPA Levels In Vivo. ACS Med. Chem. Lett..

[4] Jones S., Ahmet J., Ayton K., Ball M., Cockerill M., Fairweather E., Hamilton N., Harper P., Hitchin J., Jordan A. (2016). Discovery and Optimization of Allosteric Inhibitors of Mutant Isocitrate Dehydrogenase 1 (R132H IDH1) Displaying Activity in Human Acute Myeloid Leukemia Cells. J. Med. Chem..

[5] Jones C.K., Sheffler D.J., Williams R., Jadhav S.B., Felts A.S., Morrison R.D., Niswender C.M., Daniels J.S., Conn P.J., Lindsley C.W. (2014). Novel GlyT1 inhibitor chemotypes by scaffold hopping. Part 1: Development of a potent and CNS penetrant [3.1.0]-based lead. Bioorg. Med. Chem. Lett..

[6] Cho H.P., Engers D.W., Venable D.F., Niswender C.M., Lindsley C.W., Conn P.J., Emmitte K.A., Rodriguez A.L. (2014). A Novel Class of Succinimide-Derived Negative Allosteric Modulators of Metabotropic Glutamate Receptor Subtype 1 Provides Insight into a Disconnect in Activity between the Rat and Human Receptors. ACS Chem. Neurosci..

[7] Xiang Z., Thompson A.D., Brogan J.T., Schulte M.L., Melancon B.J., Mi D., Lewis L.M., Zou B., Yang L., Morrison R. (2011). The Discovery and Characterization of ML218: A Novel, Centrally Active T-Type Calcium Channel Inhibitor with Robust Effects in STN Neurons and in a Rodent Model of Parkinson’s Disease. ACS Chem. Neurosci..

[8] Robertson W.M., Kastrinsky D.B., Hwang I., Boger D.L. (2010). Synthesis and evaluation of a series of C5′-substituted duocarmycin SA analogs. Bioorg. Med. Chem. Lett..

[9] Anquetin G., Rouquayrol M., Mahmoudi N., Santillana-Hayat M., Gozalbes R., Greiner J., Farhati K., Derouin F., Guedj R., Vierling P. (2004). Synthesis of new fluoroquinolones and evaluation of their in vitro activity on Toxoplasma gondii and *Plasmodium* spp.. Bioorg. Med. Chem. Lett..

[10] Chanda P.B., Boyle K.E., Brody D.M., Shukla V., Boger D.L. (2016). Synthesis and evaluation of duocarmycin SA analogs incorporating the methyl 1,2,8,8a-tetrahydrocyclopropa[*c*]imidazolo [4,5-*e*]indol-4-one-6-carboxylate (CImI) alkylation subunit. Bioorg. Med. Chem..

[11] Su P., Li H., Chen W., Luo G., Yang G., Chai Z. (2020). Lewis Acid Catalyzed [3+2] Annulations of γ-Butyrolactam-Fused Donor-Acceptor Cyclopropanes with Aromatic Aldehydes and Aldimines. Eur. J. Org. Chem..

[12] Karadsheh R., Meuser M.E., Cocklin S. (2020). Composition and orientation of the core region of novel HIV-1 entry inhibitors influences metabolic stability. Molecules.

[13] Časar Z. (2020). Synthetic Approaches to Contemporary Drugs that Contain the Cyclopropyl Moiety. Synthesis.

[14] Lee M., Adams A., Cox P.B., Sanford M.S. (2019). Access to 3D Alicyclic Amine-Containing Fragments through Transannular C-H Arylation. Synlett.

[15] Harris M.R., Li Q., Lian Y., Xiao J., Londregan A.T. (2017). Construction of 1-Heteroaryl-3-azabicyclo[3.1.0]hexanes by sp^3^-sp^2^ Suzuki-Miyaura and Chan-Evans-Lam Coupling Reactions of Tertiary Trifluoroborates. Org. Lett..

[16] Topczewski J.J., Cabrera P.J., Saper N.I., Sanford M.S. (2016). Palladium-catalysed transannular C-H functionalization of alicyclic amines. Nature.

[17] Zapol’skii V.A., Namyslo J.C., de Meijere A., Kaufmann D.E. (2012). Chemistry of polyhalogenated nitrobutadienes, 10: Synthesis of highly functionalized heterocycles with a rigid 6-amino-3-azabicyclo[3.1.0]hexane moiety. Beilstein J. Org. Chem..

[18] Ren Z., Cao W., Lu Y., Wang Y., Wang S. (2008). Ring-expansion reaction of cyclopropane: A novel process for synthesis of bicyclic dicarboximides from cyclopropanedicarboximides and carbon nucleophile. Synth. Commun..

[19] Adams D.J., Blake A.J., Cooke P.A., Gill C.D., Simpkins N.S. (2002). Highly enantioselective synthesis of chiral imides and derived products via chiral base desymmetrization. Tetrahedron.

[20] Shen W.-B., Tang X.-T., Zhang T.-T., Lv D.-C., Zhao D., Su T.-F., Meng L. (2021). Copper(I)-Catalyzed Enyne Oxidation/Cyclopropanation: Divergent and Enantioselective Synthesis of Cyclopropanes. Org. Lett..

[21] Shcherbakov N.V., Dar’in D.V., Kukushkin V.Y., Dubovtsev A.Y. (2021). Gold-Catalyzed Nitrene Transfer from Benzofuroxans to N-Allylynamides: Synthesis of 3-Azabicyclo[3.1.0]hexanes. J. Org. Chem..

[22] Ito T., Takenaka H., Homma H., Harada S., Nemoto T. (2021). Stereoselective construction of fused cyclopropane from ynamide and its application to synthesis of small drug candidate molecules. Tetrahedron Lett..

[23] Murai M., Taniguchi R., Takai K. (2020). Cyclization of 1,n-Enynes Initiated by the Addition Reaction of gem-Dichromiomethane Reagents to Alkynes. Org. Lett..

[24] Liu X., Wang Z.-S., Zhai T.-Y., Luo C., Zhang Y.-P., Chen Y.-B., Deng C., Liu R.-S., Ye L.-W. (2020). Copper-Catalyzed Azide-Ynamide Cyclization to Generate α-Imino Copper Carbenes: Divergent and Enantioselective Access to Polycyclic N-Heterocycles. Angew. Chem. Int. Ed..

[25] Gao M., Gao Q., Hao X., Wu Y., Zhang Q., Liu G., Liu R. (2020). Ruthenium Carbene-Mediated Construction of Strained Allenes via the Enyne Cross-Metathesis/Cyclopropanation of 1,6-Enynes. Org. Lett..

[26] Tian X., Song L., Rudolph M., Rominger F., Oeser T., Hashmi A.S.K. (2019). Sulfilimines as Versatile Nitrene Transfer Reagents: Facile Access to Diverse Aza-Heterocycles. Angew. Chem. Int. Ed..

[27] Song L., Tian X., Rudolph M., Rominger F., Hashmi A.S.K. (2019). Gold(III)-catalyzed chemoselective annulations of anthranils with *N*-allylynamides for the synthesis of 3-azabicyclo[3.1.0]hexan-2-imines. Chem. Commun..

[28] Adcock H.V., Chatzopoulou E., Davies P.W. (2015). Divergent C-H Insertion-Cyclization Cascades of *N*-Allyl Ynamides. Angew. Chem. Int. Ed..

[29] Arumugam K., Varghese B., Brantley J.N., Konda S.S.M., Lynch V.M., Bielawski C.W. (2014). 1,6-Enyne Cyclizations Catalyzed by *N*-Heterocyclic Carbene Supported Gold Complexes: Deconvoluting Sterics and Electronics. Eur. J. Org. Chem..

[30] Liu R., Winston-McPherson G.N., Yang Z.-Y., Zhou X., Song W., Guzei I.A., Xu X., Tang W. (2013). Generation of Rhodium(I) Carbenes from Ynamides and Their Reactions with Alkynes and Alkenes. J. Am. Chem. Soc..

[31] Huang X., Klimczyk S., Veiros L.F., Maulide N. (2013). Stereoselective intramolecular cyclopropanation through catalytic olefin activation. Chem. Sci..

[32] Amijs C.H.M., Ferrer C., Echavarren A.M. (2007). Gold(I)-catalysed arylation of 1,6-enynes: Different site reactivity of cyclopropyl gold carbenes. Chem. Commun..

[33] Richter M.J.R., Zecri F.J., Briner K., Schreiber S.L. (2022). Modular Synthesis of Cyclopropane-Fused *N*-Heterocycles Enabled by Underexplored Diazo Reagents. Angew. Chem. Int. Ed. Engl..

[34] Ide K., Furuta M., Tokuyama H. (2021). Photoredox-catalyzed intramolecular cyclopropanation of alkenes with α-bromo-β-keto esters. Org. Biomol. Chem..

[35] Deng Y., Zhang J., Bankhead B., Markham J.P., Zeller M. (2021). Photoinduced oxidative cyclopropanation of ene-ynamides: Synthesis of 3-aza[n.1.0]bicycles via vinyl radicals. Chem. Commun..

[36] Muriel B., Gagnebin A., Waser J. (2019). Synthesis of bicyclo[3.1.0]hexanes by (3 + 2) annulation of cyclopropenes with aminocyclopropanes. Chem. Sci..

[37] Abbasi Kejani A., Khosravi H., Rominger F., Balalaie S., Breit B. (2021). Metal-Free Domino Oligocyclization Reactions of Enynals and Enynones with Molecular Oxygen. Org. Lett..

[38] Mutra M.R., Dhandabani G.K., Li J., Wang J.-J. (2020). Regio- and chemoselective synthesis of nitrogen-containing heterocycles via the oxidative cascade cyclization of unactivated 1,n-enynes. Chem. Commun..

[39] Jia P., Zhang Q., Zhuge Y., Liwei X., Huang Y. (2018). One-Pot Synthesis of Cyclopropanes from Methylene Azabicyclo[3.1.0]hexanes Obtained by Formal Sequential [1 + 2]- and [2 + 3]-Cycloaddition Reaction of Prop-2-ynylsulfonium Salts and Tosylaminomethyl Enones. Adv. Synth. Catal..

[40] Luo C., Wang Z., Huang Y. (2015). Asymmetric intramolecular α-cyclopropanation of aldehydes using a donor/acceptor carbene mimetic. Nat. Commun..

[41] Hansmann M.M., Melen R.L., Rudolph M., Rominger F., Wadepohl H., Stephan D.W., Hashmi A.S.K. (2015). Cyclopropanation/Carboboration Reactions of Enynes with B(C_6_F_5_)3. J. Am. Chem. Soc..

[42] Fritz S.P., Matlock J.V., McGarrigle E.M., Aggarwal V.K. (2013). Efficient Synthesis of Cyclopropane-Fused Heterocycles with Bromoethylsulfonium Salt. Chem. Eur. J..

[43] Pons A., Decaens J., Najjar R., Otog N., Arribat M., Jolly S., Couve-Bonnaire S., Sebban M., Coadou G., Oulyadi H. (2022). Fluorocyclopropane-Containing Proline Analogue: Synthesis and Conformation of an Item in the Peptide Chemist’s Toolbox. ACS Omega.

[44] Ohsawa K., Kubota J., Ochiai S., Doi T. (2021). Stereoselective Synthesis of 1-Aminocyclopropanecarboxylic Acid Carnosadines via Inter-intramolecular Double Alkylation with Optically Active 2-Methylaziridine Derivatives. J. Org. Chem..

[45] Maolanon A., Papangelis A., Kawiecki D., Mou T.-C., Syrenne J.T., Yi F., Hansen K.B., Clausen R.P. (2021). Stereoselective synthesis of novel 2′-(S)-CCG-IV analogs as potent NMDA receptor agonists. Eur. J. Med. Chem..

[46] Zhuang Z., Yu J.-Q. (2020). Pd(II)-Catalyzed Enantioselective γ-C(sp3)-H Functionalizations of Free Cyclopropylmethylamines. J. Am. Chem. Soc..

[47] Yuan Y., Zheng Z.-J., Ye F., Ma J.-H., Xu Z., Bai X.-F., Li L., Xu L.-W. (2018). Highly efficient desymmetrization of cyclopropenes to azabicyclo[3.1.0]hexanes with five continuous stereogenic centers by copper-catalyzed [3 + 2] cycloadditions. Org. Chem. Front..

[48] Maslivetc V., Barrett C., Aksenov N.A., Rubina M., Rubin M. (2018). Intramolecular nucleophilic addition of carbanions generated from *N*-benzylamides to cyclopropenes. Org. Biomol. Chem..

[49] Pedroni J., Cramer N. (2017). Enantioselective C-H Functionalization-Addition Sequence Delivers Densely Substituted 3-Azabicyclo[3.1.0]hexanes. J. Am. Chem. Soc..

[50] McCabe S.R., Wipf P. (2017). Eight-Step Enantioselective Total Synthesis of (-)-Cycloclavine. Angew. Chem. Int. Ed..

[51] Petronijevic F.R., Wipf P. (2011). Total Synthesis of (±)-Cycloclavine and (±)-5-epi-Cycloclavine. J. Am. Chem. Soc..

[52] Wasa M., Engle K.M., Yu J.-Q. (2010). Pd(II)-Catalyzed Olefination of sp3 C-H Bonds. J. Am. Chem. Soc..

[53] Maximiano A.P., Ramos G.S., Marques M.V., Sá M.M. (2021). Functionalized Cyclopropanes as Versatile Intermediates for the Diversity-Oriented Synthesis of γ-Lactones, γ-Lactams and δ-Lactams. Synthesis.

[54] Kleban I., Krokhmaliuk Y., Reut S., Shuvakin S., Pendyukh V.V., Khyzhan O.I., Yarmoliuk D.S., Tymtsunik A.V., Rassukana Y.V., Grygorenko O.O. (2021). Multigram Synthesis of Heterabicyclo[n.1.0]alkan-1-yl Trifluoroborates. Eur. J. Org. Chem..

[55] Liu L., Shao L., Li J., Cui H., Li B., Zhou X., Lv P., Zhang J. (2019). Synthesis, antibacterial activities, mode of action and acute toxicity studies of new oxazolidinone-fluoroquinolone hybrids. Molecules.

[56] Zheng Y., Yu X., Lv S., Mykhailiuk P.K., Ma Q., Hai L., Wu Y. (2018). Synthesis of CHF_2_-substituted 3-azabicyclo[3.1.0]hexanes by photochemical decomposition of CHF_2_-pyrazolines. RSC Adv..

[57] Bychek R.M., Levterov V.V., Sadkova I.V., Tolmachev A.A., Mykhailiuk P.K. (2018). Synthesis of Functionalized Difluorocyclopropanes: Unique Building Blocks for Drug Discovery. Chem. Eur. J..

[58] Chen P., Zhu C., Zhu R., Lin Z., Wu W., Jiang H. (2017). Synthesis of 3-azabicyclo[3.1.0]hexane derivatives via palladium-catalyzed cyclopropanation of maleimides with *N*-tosylhydrazones: Practical and facile access to CP-866,087. Org. Biomol. Chem..

[59] Manna S., Antonchick A.P. (2015). Copper-catalyzed (2 + 1) annulation of acetophenones with maleimides: Direct synthesis of cyclopropanes. Angew. Chem. Int. Ed..

[60] Gomes A.T.P.C., Faustino M.A.F., Neves M.G.P.M.S., Ferreira V.F., Juarranz A., Cavaleiro J.A.S., Sanz-Rodriguez F. (2015). Photodynamic effect of glycochlorin conjugates in human cancer epithelial cells. RSC Adv..

[61] Vempati R.K., Reddy N.S., Alapati S.R., Dubey P.K. (2013). Synthesis of azabicyclo[3.1.0]amine analogues of anacardic acid as potent antibacterial agents. Asian J. Chem..

[62] Oikawa M., Sasaki S., Sakai M., Ishikawa Y., Sakai R. (2012). Total Synthesis of (±)-Dysibetaine CPa and Analogs. Eur. J. Org. Chem..

[63] Louhichi N., Haouas A., Ben Hamadi N., Msaddek M. (2011). Synthesis and chemistry of pyrazolines derived from diphenyldiazomethane. Heterocycl. Commun..

[64] Mykhailiuk P.K., Afonin S., Palamarchuk G.V., Shishkin O.V., Ulrich A.S., Komarov I.V. (2008). Synthesis of trifluoromethyl-substituted proline analogues as ^19^F NMR labels for peptides in the polyproline II conformation. Angew. Chem. Int. Ed..

[65] Schmidt M.A., Simmons E.M., Wei C.S., Park H., Eastgate M.D. (2018). An Enantioselective Total Synthesis of (+)-Duocarmycin SA. J. Org. Chem..

[66] Uematsu M., Brody D.M., Boger D.L. (2015). A five-membered lactone prodrug of CBI-based analogs of the duocarmycins. Tetrahedron Lett..

[67] Tercel M., Pruijn F.B., O’Connor P.D., Liyanage H.D.S., Atwell G.J., Alix S.M. (2014). Mechanism of Action of AminoCBIs: Highly Reactive but Highly Cytotoxic Analogues of the Duocarmycins. ChemBioChem.

[68] MacMillan K.S., Nguyen T., Hwang I., Boger D.L. (2009). Total Synthesis and Evaluation of iso-Duocarmycin SA and iso-Yatakemycin. J. Am. Chem. Soc..

[69] Gauss C.M., Hamasaki A., Parrish J.P., MacMillan K.S., Rayl T.J., Hwang I., Boger D.L. (2009). Synthesis and preliminary evaluation of duocarmycin analogues incorporating the 1,2,11,11a-tetrahydrocyclopropa[*c*]naphtho[2,3-*e*]indol-4-one (CNI) and 1,2,11,11a-tetrahydrocyclopropa[*c*]naphtho[1,2-*e*]indol-4-one (*iso*-CNI) alkylation subunits. Tetrahedron.

[70] MacMillan K.S., Boger D.L. (2008). An Additional Spirocyclization for Duocarmycin SA. J. Am. Chem. Soc..

[71] Tichenor M.S., MacMillan K.S., Stover J.S., Wolkenberg S.E., Pavani M.G., Zanella L., Zaid A.N., Spalluto G., Rayl T.J., Hwang I. (2007). Rational Design, Synthesis, and Evaluation of Key Analogues of CC-1065 and the Duocarmycins. J. Am. Chem. Soc..

[72] Tichenor M.S., Trzupek J.D., Kastrinsky D.B., Shiga F., Hwang I., Boger D.L. (2006). Asymmetric Total Synthesis of (+)- and ent-(−)-Yatakemycin and Duocarmycin SA. Evaluation of Yatakemycin Key Partial Structures and Its Unnatural Enantiomer. J. Am. Chem. Soc..

[73] Tietze L.F., Herzig T., Feuerstein T., Schuberth I. (2002). Synthesis and biological evaluation of novel analogues and prodrugs of the cytotoxic antibiotic CC-1065 for selective cancer therapy. Eur. J. Org. Chem..

[74] Amishiro N., Nagamura S., Kobayashi E., Okamoto A., Gomi K., Saito H. (1999). Synthesis and antitumor activity of duocarmycin derivatives: A-ring pyrrole compounds bearing 5-membered heteroarylacryloyl groups. Chem. Pharm. Bull..

[75] Nagamura S., Asai A., Kanda Y., Kobayashi E., Gomi K., Saito H. (1996). Synthesis and antitumor activity of duocarmycin derivatives: Modification of segment A of duocarmycin B2. Chem. Pharm. Bull..

[76] Yasuzawa T., Muroi K., Ichimura M., Takahashi I., Ogawa T., Takahashi K., Sano H., Saitoh Y. (1995). Duocarmycins, potent antitumor antibiotics produced by *Streptomyces* sp. structures and chemistry. Chem. Pharm. Bull..

[77] Tietze L.F., Grote T. (1993). Synthesis of the reduced A-unit (CI) of the antitumor antibiotic CC-1065. Chem. Ber..

[78] Jing Z., Wang G., Zhou Y., Pang D., Zhu F., Liu H. (2020). Selectivity of 18-crown-6 ether to alkali ions by density functional theory and molecular dynamics simulation. J. Mol. Liq..

[79] Pudlo M., Csányi D., Moreau F., Hajós G., Riedl Z., Sapi J. (2007). First Suzuki-Miyaura type cross-coupling of *ortho*-azidobromobenzene with arylboronic acids and its application to the synthesis of fused aromatic indole-heterocycles. Tetrahedron.

[80] Zhao Y.-L., Li Y., Li S.-M., Zhou Y.-G., Sun F.-Y., Gao L.-X., Han F.-S. (2011). A Highly Practical and Reliable Nickel Catalyst for Suzuki-Miyaura Coupling of Aryl Halides. Adv. Synth. Catal..

[81] Wang X., Zhang S., Cui P., Li S. (2020). Modular Synthesis of Drimane Meroterpenoids Leveraging Decarboxylative Borylation and Suzuki Coupling. Org. Lett..

[82] Bai Y., Li X., Xiao X., Liu J., Yang J., Wang J. (2017). Synthesis of Imidazolium Precursors for the Hydroxyl-Group-Modified *N*-Heterocyclic Carbenes and Applications of the in situ Generated Carbene Ligands in Suzuki-Miyaura and Sonogashira Coupling Reactions. Chin. J. Org. Chem..

[83] Sharma N., Li Z., Sharma U.K., Van der Eycken E.V. (2014). Facile Access to Functionalized Spiro[indoline-3,2′-pyrrole]-2,5′-diones via Post-Ugi Domino Buchwald-Hartwig/Michael Reaction. Org. Lett..

[84] Butani H.H., Vachhani D.D., Bhoya U.C., Shah A.K., Van der Eycken E.V. (2014). Regio- and Chemoselective Formation of Spiroindolinone-Isoindolinone by a Palladium-Catalyzed Buchwald-Hartwig Addition-Elimination Sequence. Eur. J. Org. Chem..

[85] Yuen O.Y., Leung M.P., So C.M., Sun R.W.-Y., Kwong F.Y. (2018). Palladium-Catalyzed Direct Arylation of Polyfluoroarenes for Accessing Tetra-ortho-Substituted Biaryls: Buchwald-type Ligand Having Complementary -PPh_2_ Moiety Exhibits Better Efficiency. J. Org. Chem..

[86] Zhang X., Qi D., Jiao C., Liu X., Zhang G. (2021). Nickel-catalyzed deaminative Sonogashira coupling of alkylpyridinium salts enabled by NN_2_ pincer ligand. Nat. Commun..

[87] Liu R.R., Wang Y.G., Li Y.L., Huang B.B., Liang R.X., Jia Y.X. (2017). Enantioselective Dearomative Difunctionalization of Indoles by Palladium-Catalyzed Heck/Sonogashira Sequence. Angew. Chem. Int. Ed..

[88] Tang T., Fei X.-D., Ge Z.-Y., Chen Z., Zhu Y.-M., Ji S.-J. (2013). Palladium-Catalyzed Carbonylative Sonogashira Coupling of Aryl Bromides via *tert*-Butyl Isocyanide Insertion. J. Org. Chem..

[89] Chinchilla R., Najera C. (2011). Recent advances in Sonogashira reactions. Chem. Soc. Rev..

[90] Chinchilla R., Nájera C. (2007). The Sonogashira Reaction:  A Booming Methodology in Synthetic Organic Chemistry. Chem. Rev..

[91] Knowe M.T., Danneman M.W., Sun S., Pink M., Johnston J.N. (2018). Biomimetic Desymmetrization of a Carboxylic Acid. J. Am. Chem. Soc..

[92] Zhang Z.G., Zhang Y.C., Huang G.Q., Zhang G.S. (2017). Organoiodine reagent-promoted intermolecular oxidative amination: Synthesis of cyclopropyl spirooxindoles. Org. Chem. Front..

[93] Lin C.-H., Pursley D., Klein J.E.M.N., Teske J., Allen J.A., Rami F., Koehn A., Plietker B. (2015). Non-decarbonylative photochemical versus thermal activation of Bu_4_N[Fe(CO)_3_(NO)] – the Fe-catalyzed Cloke–Wilson rearrangement of vinyl and arylcyclopropanes. Chem. Sci..

