# Supplementary material for: Base-Promoted Intramolecular Addition of Vinyl Cyclopropanecarboxamides to Access Conformationally Restricted Aza[3.1.0]bicycles
Source: Molecules. 2023 Apr 25;28(9):3691. doi: 10.3390/molecules28093691 (PMC10179847; doi:10.3390/molecules28093691)

Supporting Information

Base-Promoted Intramolecular Addition of Vinyl Cyclopropanecarboxamides to Access Conformationally Restricted Aza[3.1.0]bicycles

Jingya Li, Zhiguo Zhang,* Liming Chen, Mengjuan Li, Xingjie Zhang, Guisheng Zhang*

Key Laboratory of Green Chemical Media and Reactions, Ministry of Education, Henan Key Laboratory of Organic Functional Molecule and Drug Innovation, Collaborative Innovation Center of Henan Province for Green Manufacturing of Fine Chemicals, School of Chemistry and Chemical Engineering, Henan Normal University, Xinxiang 453007, China

E-mail: zhangzg@htu.edu.cn and zgs@htu.edu.cn Fax: (+86)-373-332-5250.

Table of Contents

I. Optimization of reaction conditions……………………………………………………S2

II. General procedures……………………………………………………………S3

III. References……………………………………………………………………S5

IV. ^{1}H and ^{13}C NMR spectra……………………………………………………S6

I. Optimization of reaction conditions

Table S1: Optimization of reaction conditions.[a]

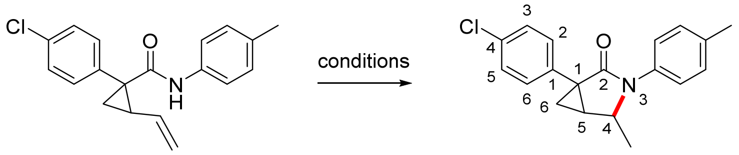

1a 2a

Entry	Base/equiv	Solvent	[O]/equiv	T/°C	Time/h	Yield/%[b]
1	^{t}BuOK (4.0)	DMF	Air	110	24	82 (11)
2	^{t}BuOK (4.0)	DMF	Air	110	24	86[c]
3	^{t}BuOK (1.0)	DMF	Air	110	24	31 (63)
4	^{t}BuOK (2.0)	DMF	Air	110	24	64 (35)
5	^{t}BuOK (3.0)	DMF	Air	110	24	72 (6)
6	^{t}BuOK (5.0)	DMF	Air	110	24	57 (26)
7	^{t}BuOK (4.0)	DMF	Air	100	24	47 (48)
8	^{t}BuOK (4.0)	DMF	Air	120	24	77 (16)
9	^{t}BuOK (4.0)	DMF	Air	130	24	73 (8)
10	^{t}BuOK (4.0)	DMF	Air	140	24	43
11	K_3PO_4 (4.0)	DMF	Air	110	24	39 (58)
12	NaH (4.0)	DMF	Air	110	24	15 (81)
13	NaOH (4.0)	DMF	Air	110	24	22 (68)
14	Cs_2CO_3(4.0)	DMF	Air	110	24	20 (72)
15	DBU (4.0)	DMF	Air	110	24	0 (93)
16	^{t}BuOK (4.0)	MeCN	Air	110	24	18 (76)
17	^{t}BuOK (4.0)	Dioxane	Air	110	24	0 (93)
18	^{t}BuOK (4.0)	Toluene	Air	110	24	0 (91)
19	^{t}BuOK (4.0)	NMP	Air	110	24	68 (6)
20	^{t}BuOK (4.0)	DMSO	Air	110	24	0 (85)

[a] Unless otherwise indicated, the reaction was conducted with **1a** (0.5 mmol, 1.0 equiv), base (4.0 equiv) and solvent (2 mL) at 110 °C under air in a sealed tube, and isolated yields are reported. [b] Recovered of **1a** was shown in parentheses. [c] in the presence of ^{t}BuOK (4.0 equiv) and 18-crown-6 ether (4.2 equiv).

II. General procedures.

Method A: General procedures for **1a-1q** (**1a** as an example)[1, 2].

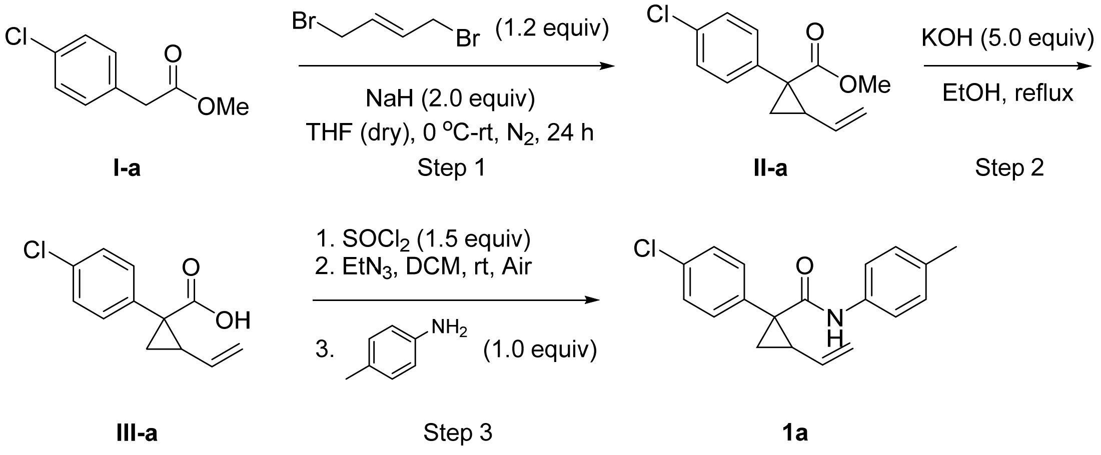

Step 1: To a flame-dried round-bottom flask under nitrogen atmosphere was added NaH (4.0 g, 100 mmol, 2.0 equiv) and THF (120 mL). The mixture was cooled to 0 ºC while stirring, upon which the methyl 2-(4-chlorophenyl)acetate **I-a** (7.7 mL, 50 mmol, 1.0 equiv) was added. The mixture was warmed to room temperature for 10 minutes then cooled to 0 ºC again. (*E*)-1,4-dibromobut-2-ene (12.8 g, 60 mmol, 1.2 equiv) was then added dropwise, and the reaction was stirred at 35 ºC for 24 hours. The reaction was quenched by addition of saturated aqueous NH_4Cl (150 mL). The layers were separated, and the aqueous layer was extracted with ethyl acetate. The organic layers were combined, dried, filtered and concentrated to an oil. The oil was purified by flash column chromatography with PE and EA (eluent: EA/PE = 1/50) as eluent to give **II-a** as a colorless liquid (8.0 g, 34 mmol, 67%).

Step 2: Methyl 1-(4-chlorophenyl)-2-vinylcyclopropane-1-carboxylate **II-a** (8.0 g, 34 mmol, 1.0 equiv), was then dissolved in EtOH (140 mL), combined with KOH (9.5 g, 170 mmol, 5.0 equiv) and stirred under reflux for 3 hours. The reaction was cooled to room temperature and acidified to pH 3-1 with 10% HCl. The aqueous layer was extracted with dichloromethane, and the combined organic layers were dried, filtered, and concentrated to obtain the target product **III-a** as a white solid. (7.54 g, 34 mmol, 99%).

Step 3: To a 50 mL round bottom flask equipped with 100 mL CH_2Cl_2 was added **III-a** (29.0 mmol, 1.0 equiv), $SOCl_2$ (3.2 mL, 43.5 mmol, 1.5 equiv), the mixture was stirred at room temperature for 10 min, then Et_3N was added dropwise to the mixture until there is no white smoke produced. After the mixture was stirred at room temperature for 2 hours, it was quenched by water, and extracted with dichloromethane (120 mL×3). The combined filtrate was washed with saturated brine (120 mL×3) and dried over with anhydrous Na_2SO_4. Then the residue was purified by a flash silica gel column chromatography (eluent: EA/PE = 1/20) to give the desired product compound **1a** as a white solid (8.2 g, 90%).

Method B: General procedure for **1r**[3].

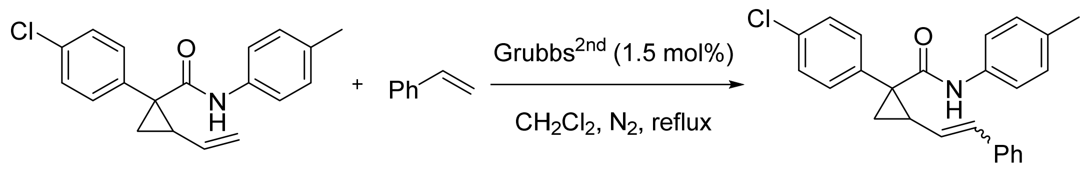

1a **1r**

A dried round-bottom flask was charged with **1a** (5.0 mmol, 1.0 equiv) and 2nd generation Grubbs catalyst (64 mg, 1.5 mol%) under an atmosphere of dry nitrogen. CH_2Cl_2 (0.6 M) was added followed by styrene (17.2 mL, 30.0 equiv). The flask was sealed under an atmosphere of dry nitrogen and the mixture stirred at 45 °C for 24 h. The solvent was removed under reduced pressure and the residue was purified by flash column chromatography with PE and EA (eluent: EA/PE = 1/40) to give the desired product compound **1r** as a white solid.

III. References

1. Knowe, M. T.; Danneman, M. W.; Sun, S.; Pink, M.; Johnston, J. N. *J. Am. Chem. Soc.* **2018**, *140,* 1998.
2. Zhang, Z. G.; Zhang, Y. C.; Huang, G. Q.; Zhang, G. S. *Org. Chem. Front.* **2017**, *4*, 1372.
3. Lin, C.-H.; Pursley, D.; Klein, J. E. M. N.; Teske, J.; Allen, J. A.; Rami, F.; Koehn, A.; Plietker, B. *Chem. Sci.* **2015**, *6*, 7034.

IV. ^{1}H NMR and ^{13}C NMR spectra

^{1}H NMR spectra of **2a** (600 MHz, $CDCl_3$)

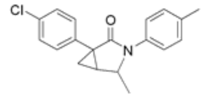

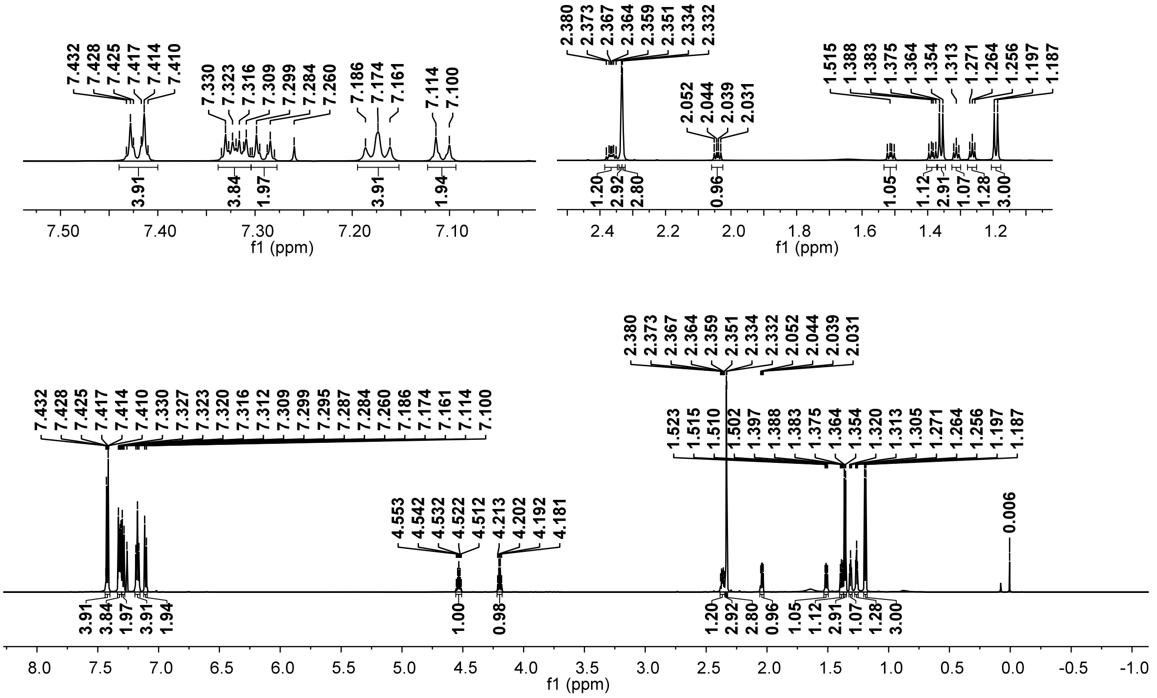

^{13}C NMR spectra of **2a** (100 MHz, $CDCl_3$)

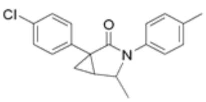

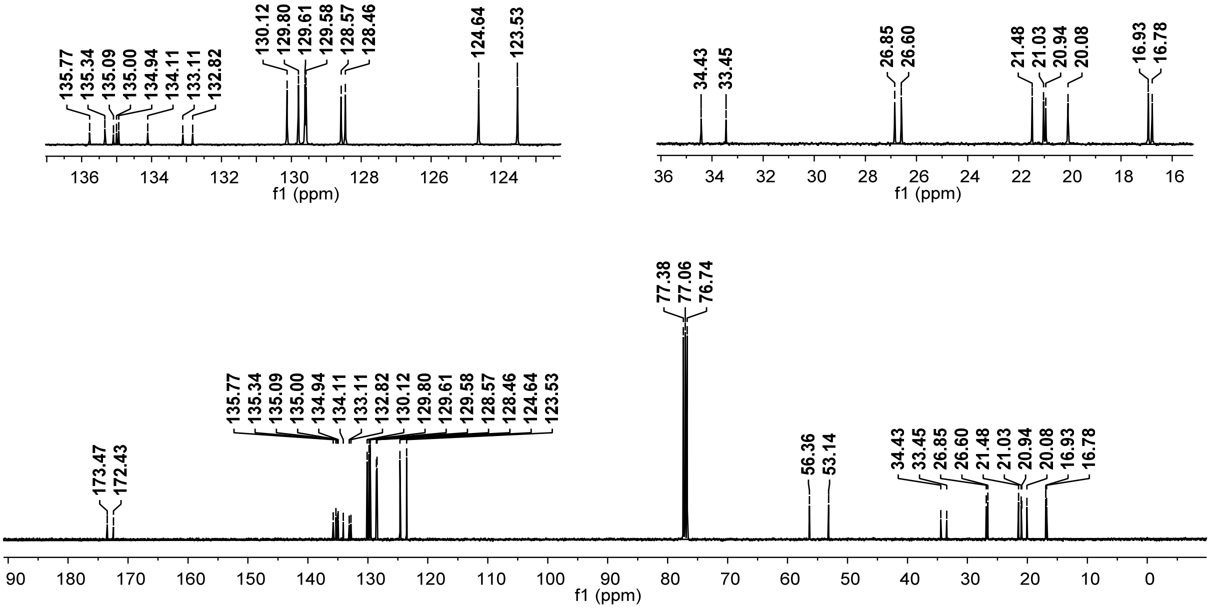

^{1}H NMR spectra of **2b** (600 MHz, $CDCl_3$)

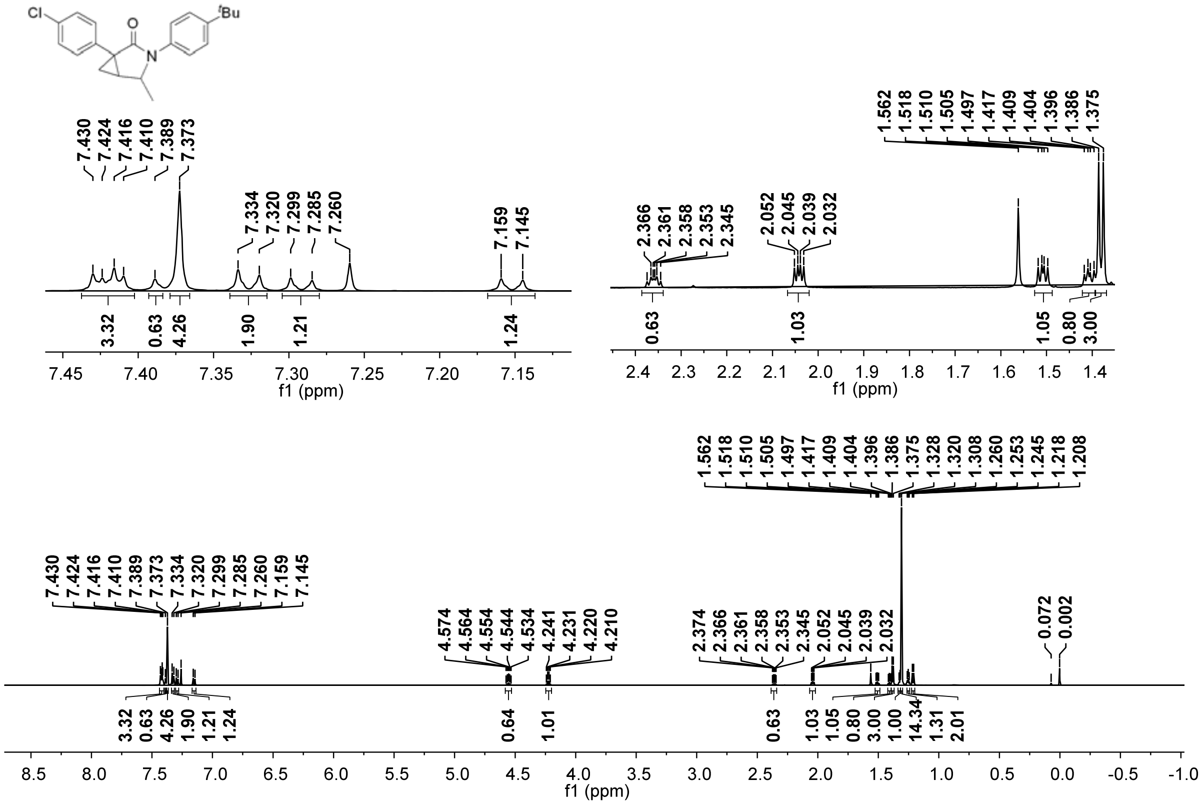

^{13}C NMR spectra of **2b** (150 MHz, $CDCl_3$)

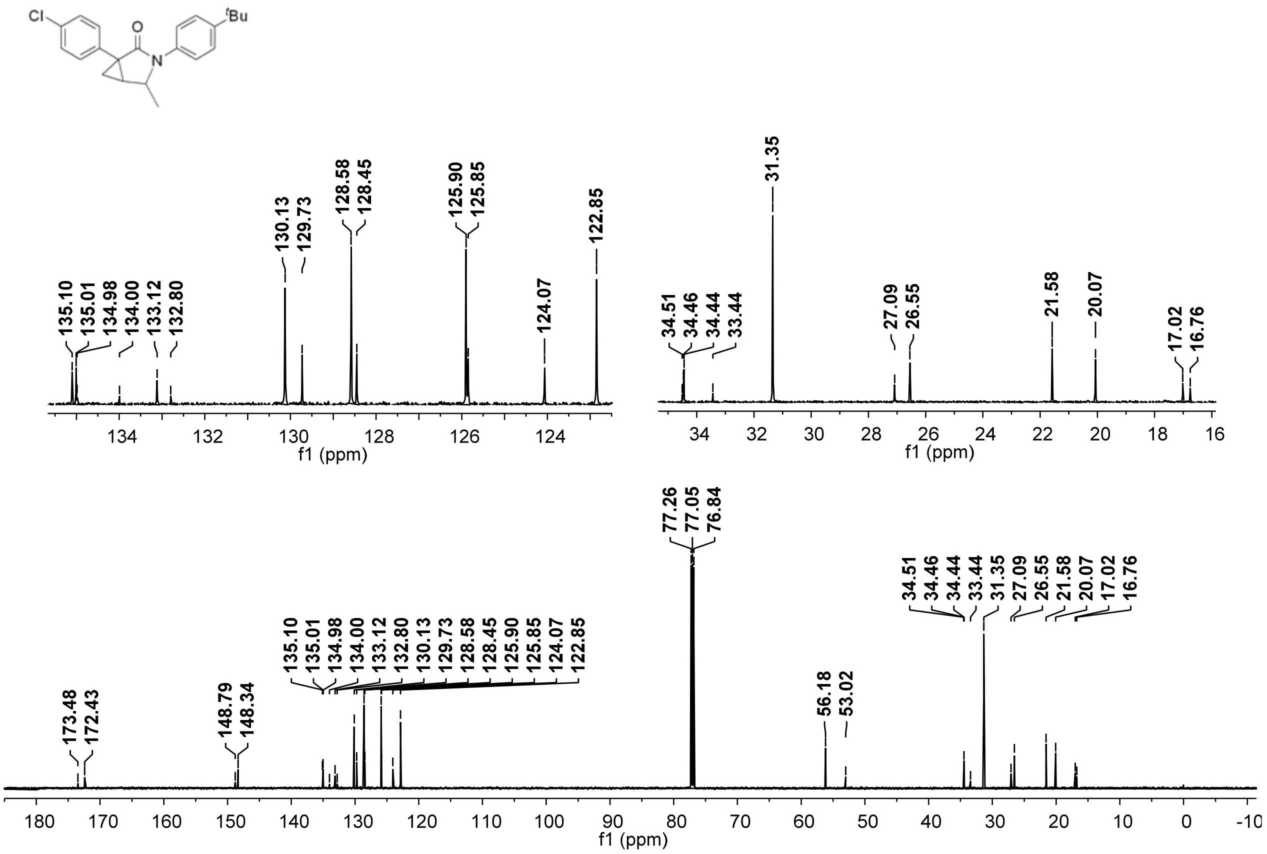

^{1}H NMR spectra of **2c** (400 MHz, $CDCl_3$)

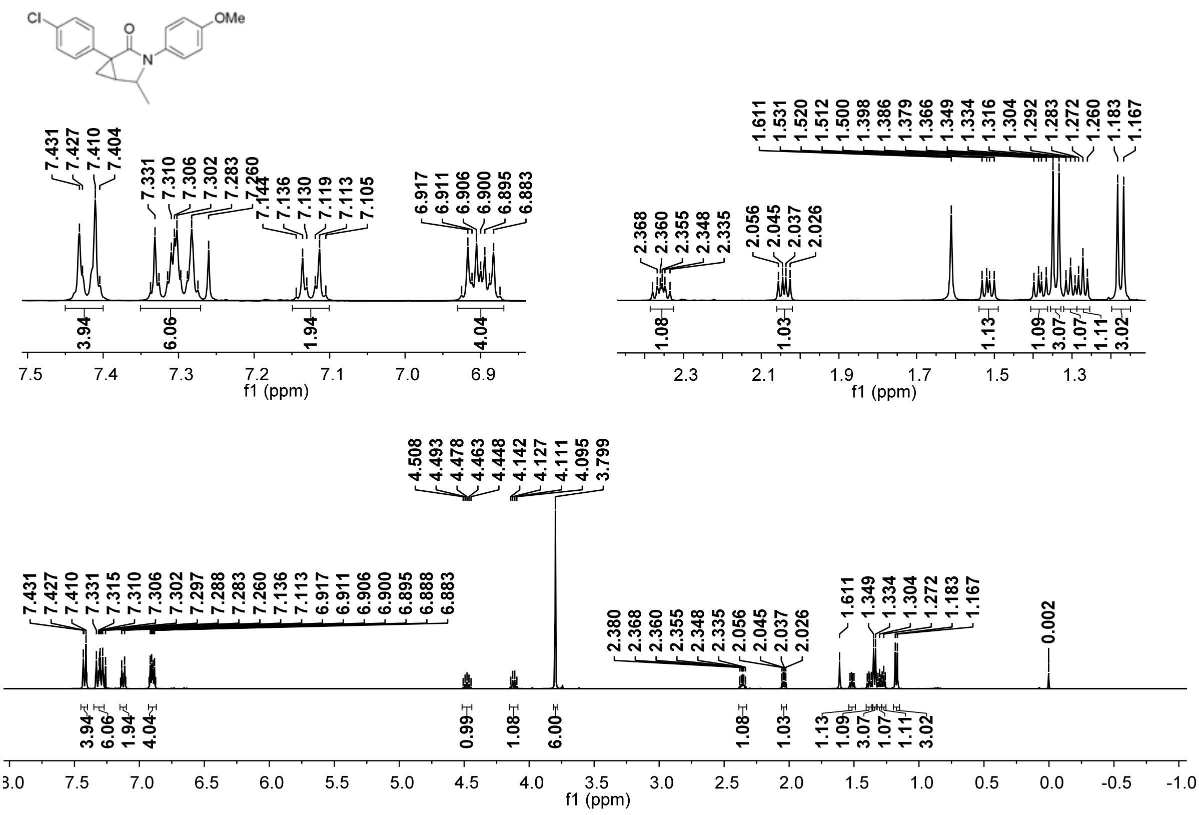

^{13}C NMR spectra of **2c** (100 MHz, $CDCl_3$)

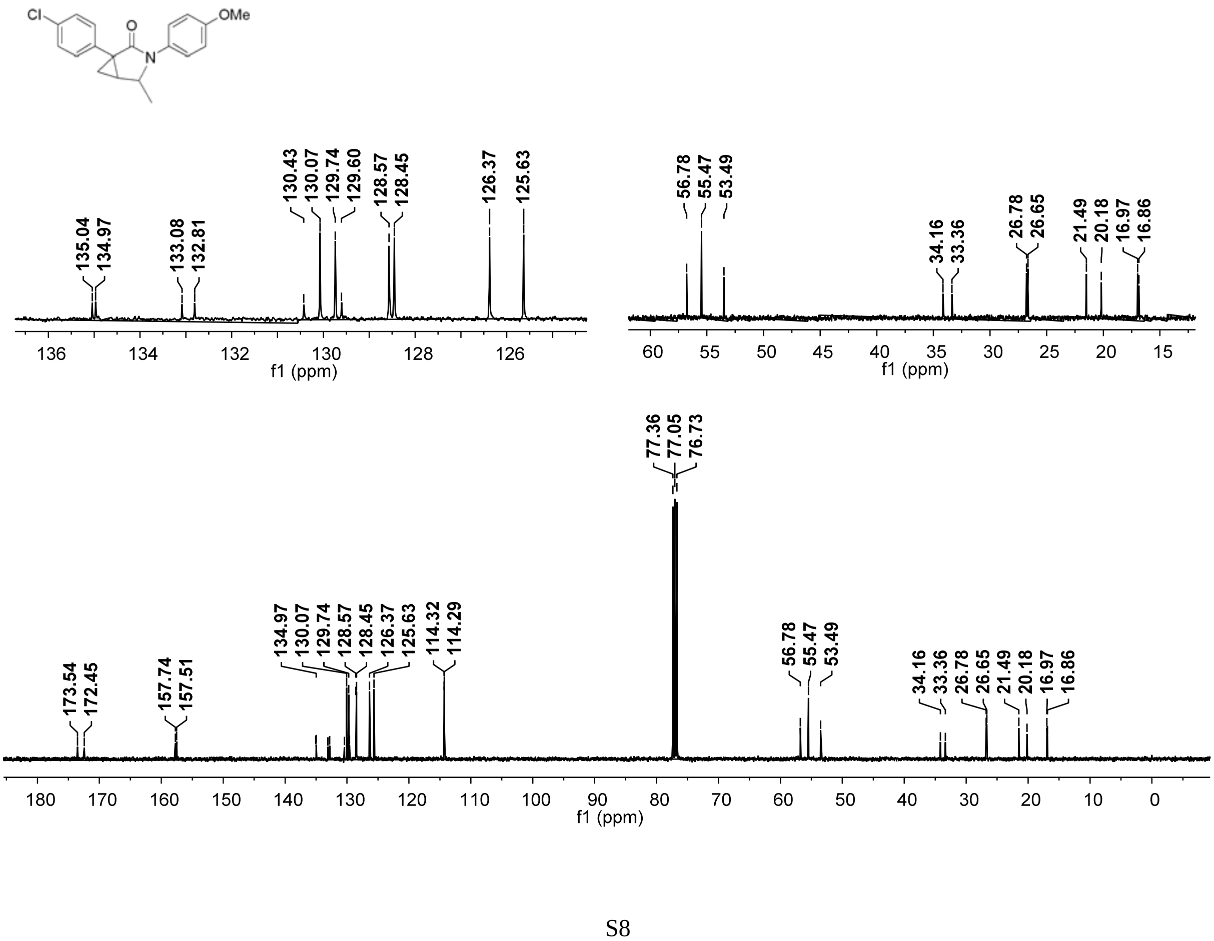

^{1}H NMR spectra of **2d** (400 MHz, $CDCl_3$)

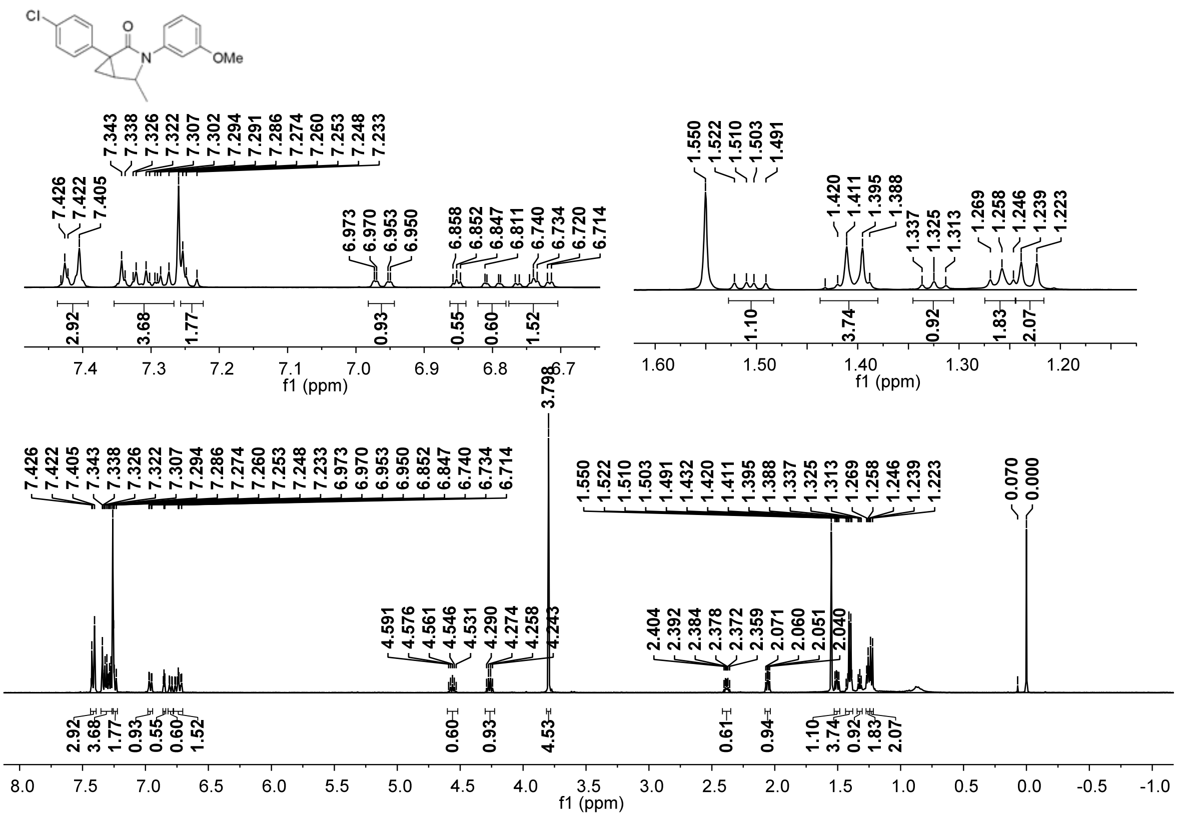

^{13}C NMR spectra of **2d** (100 MHz, $CDCl_3$)

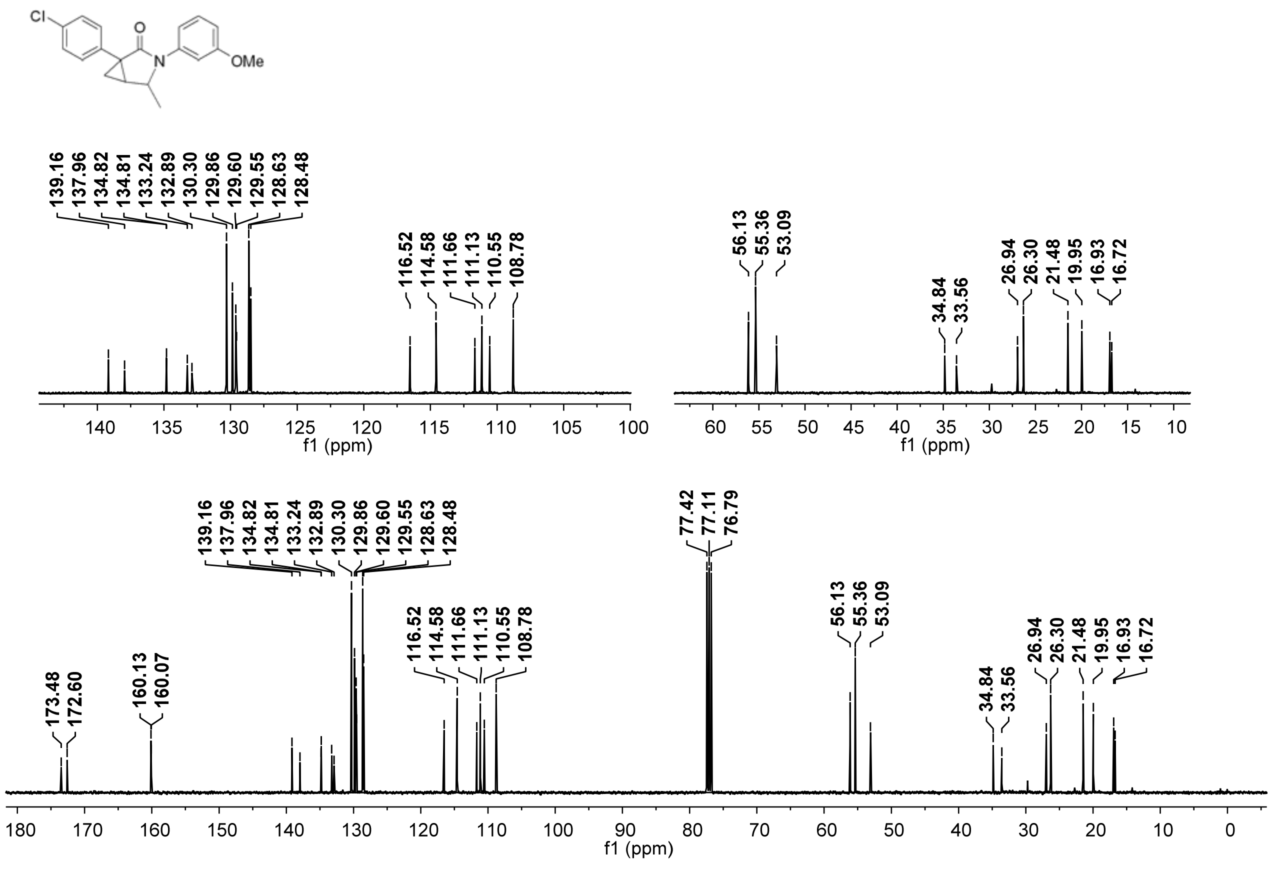

^{1}H NMR spectra of **2e** (400 MHz, $CDCl_3$)

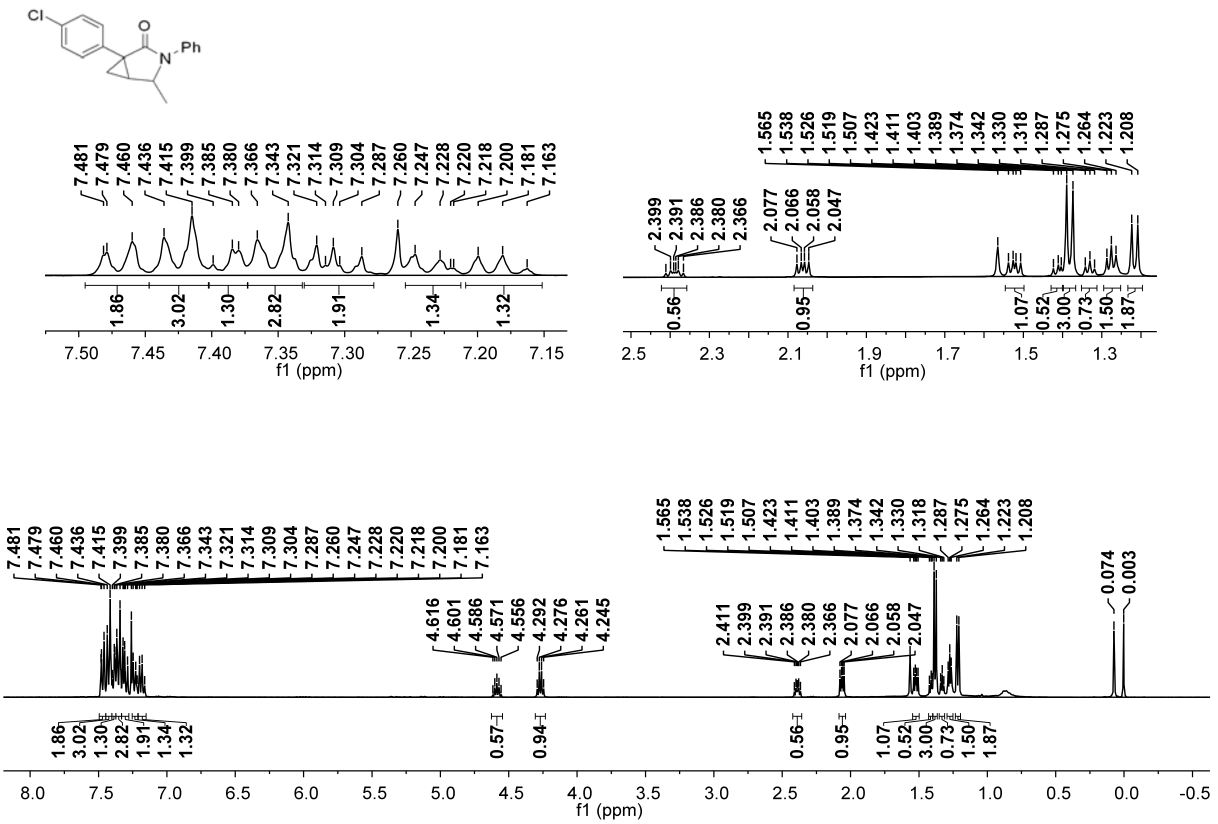

^{13}C NMR spectra of **2e** (150 MHz, $CDCl_3$)

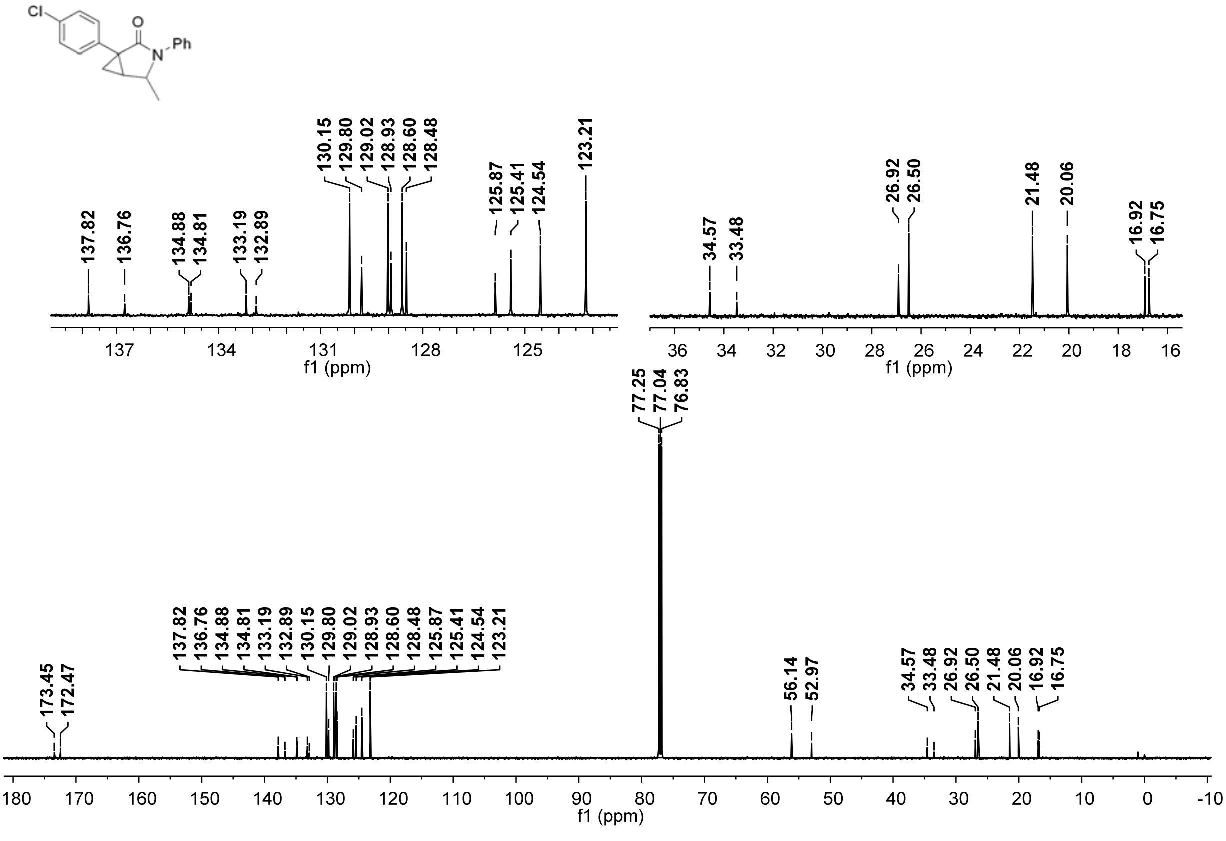

^{1}H NMR spectra of **2f** (400 MHz, $CDCl_3$)

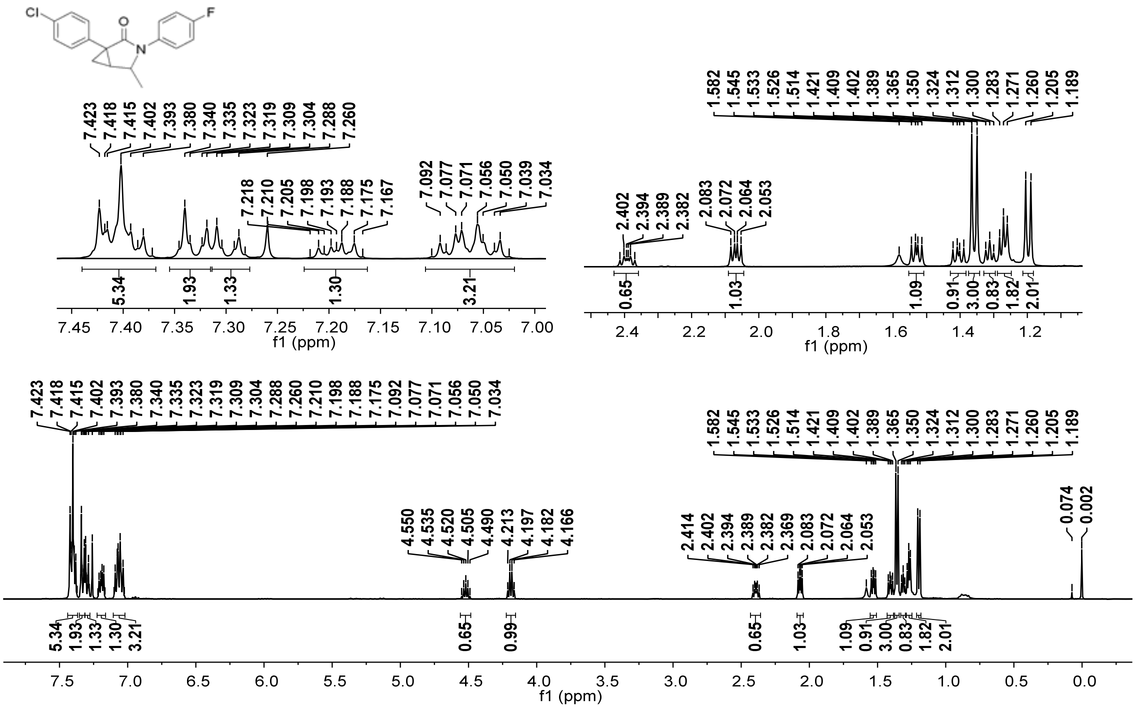

^{13}C NMR spectra of **2f** (150 MHz, $CDCl_3$)

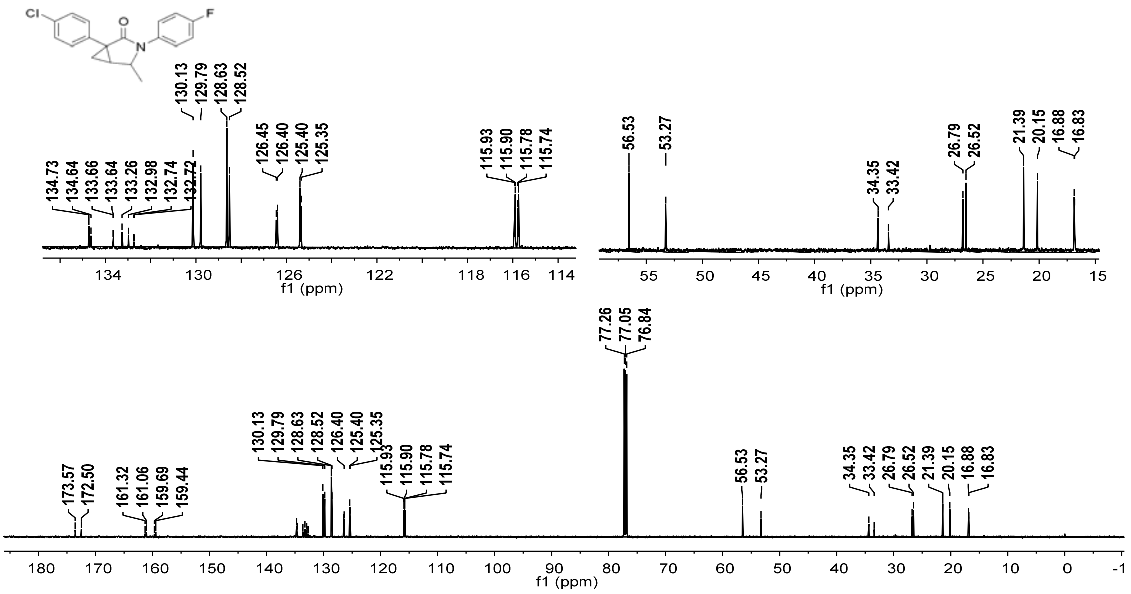

^{19}F NMR spectra of **2f** (565 MHz, $CDCl_3$)

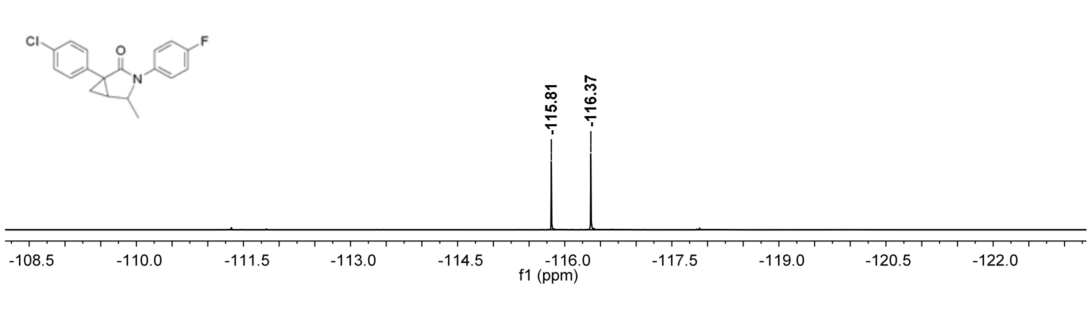

^{1}H NMR spectra of **2g** (400 MHz, $CDCl_3$)

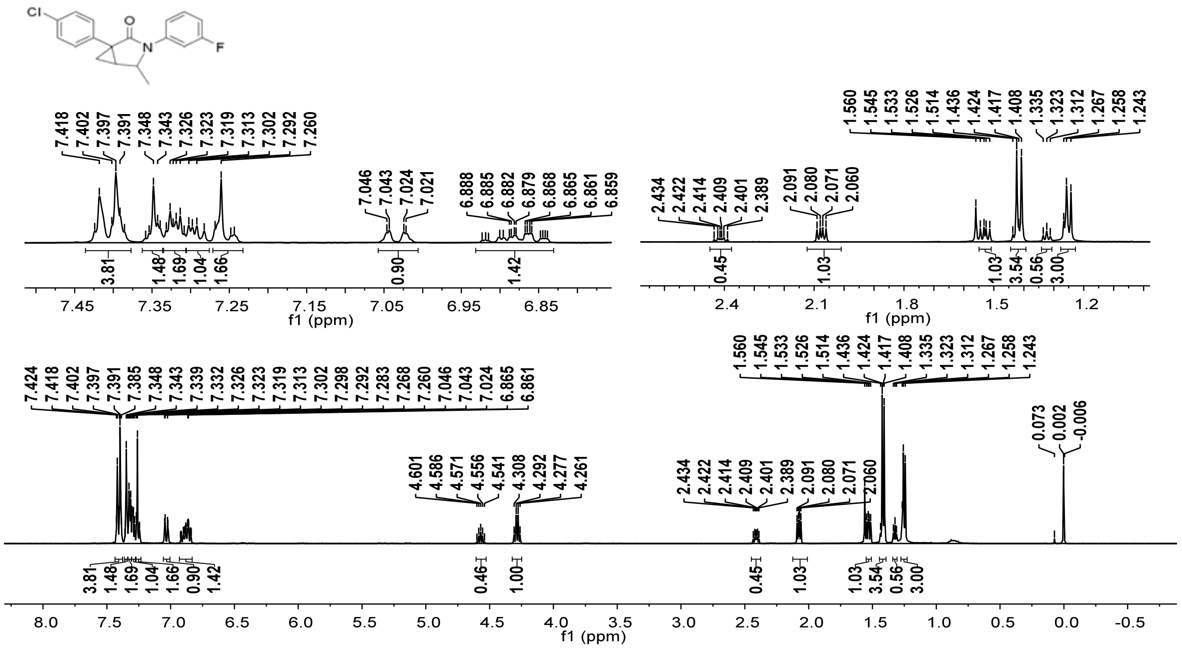

^{13}C NMR spectra of **2g** (150 MHz, $CDCl_3$)

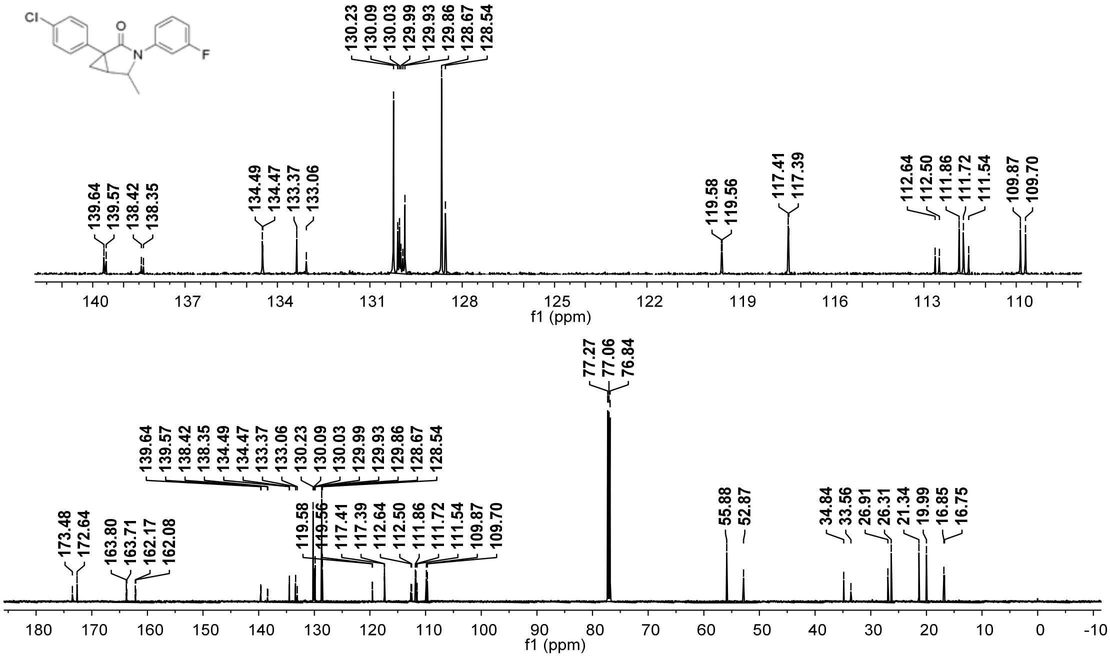

^{19}F NMR spectra of **2g** (565 MHz, $CDCl_3$)

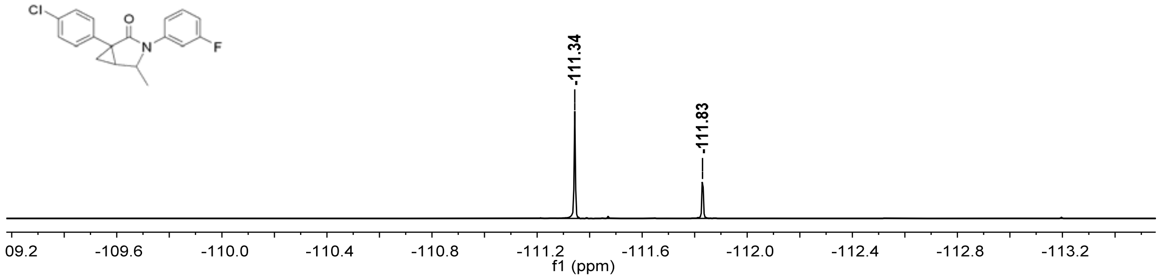

^{1}H NMR spectra of **2i** (600 MHz, $CDCl_3$)

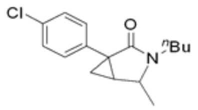

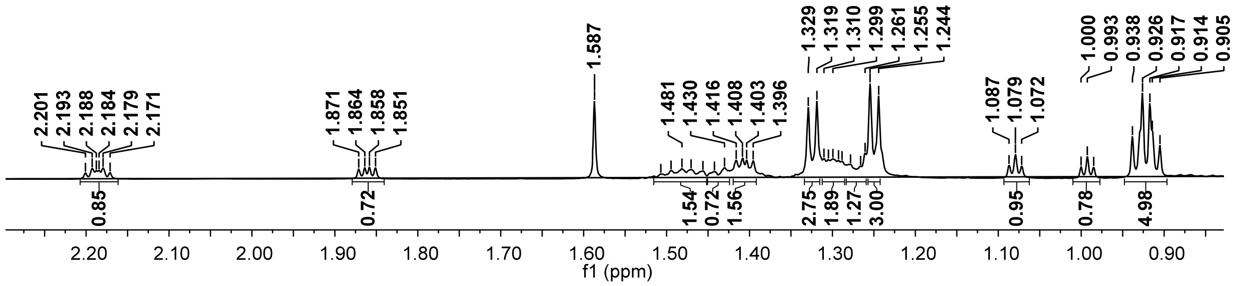

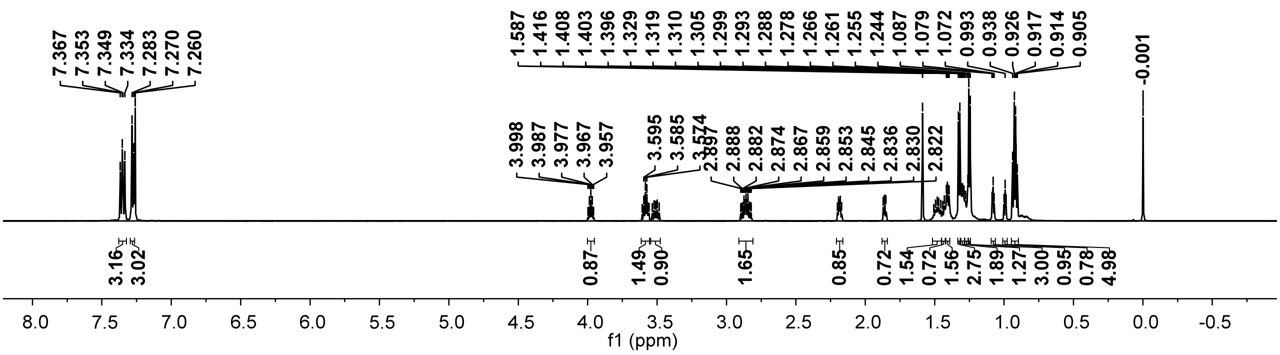

^{13}C NMR spectra of **2i** (150 MHz, $CDCl_3$)

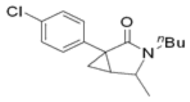

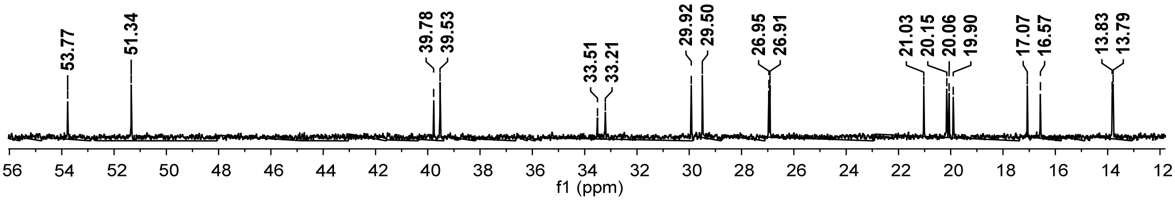

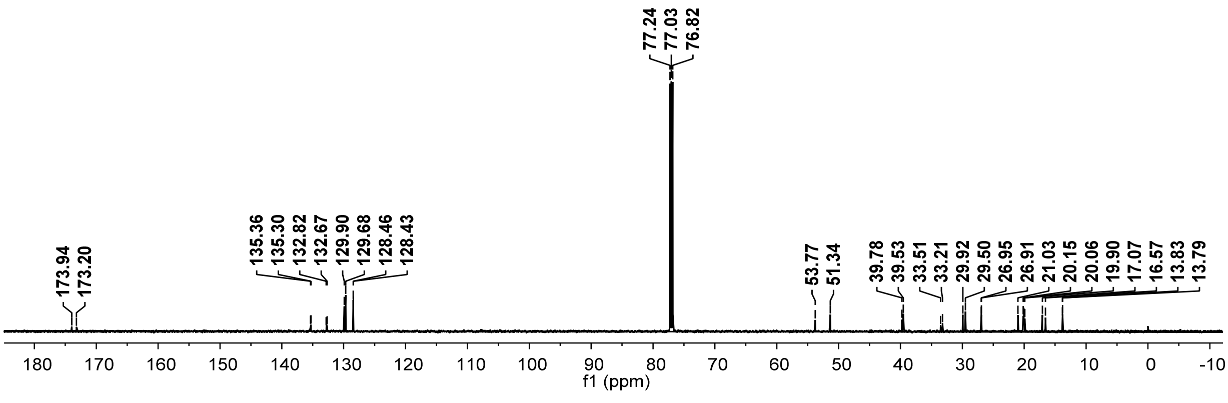

^{1}H NMR spectra of **2j** (400 MHz, $CDCl_3$)

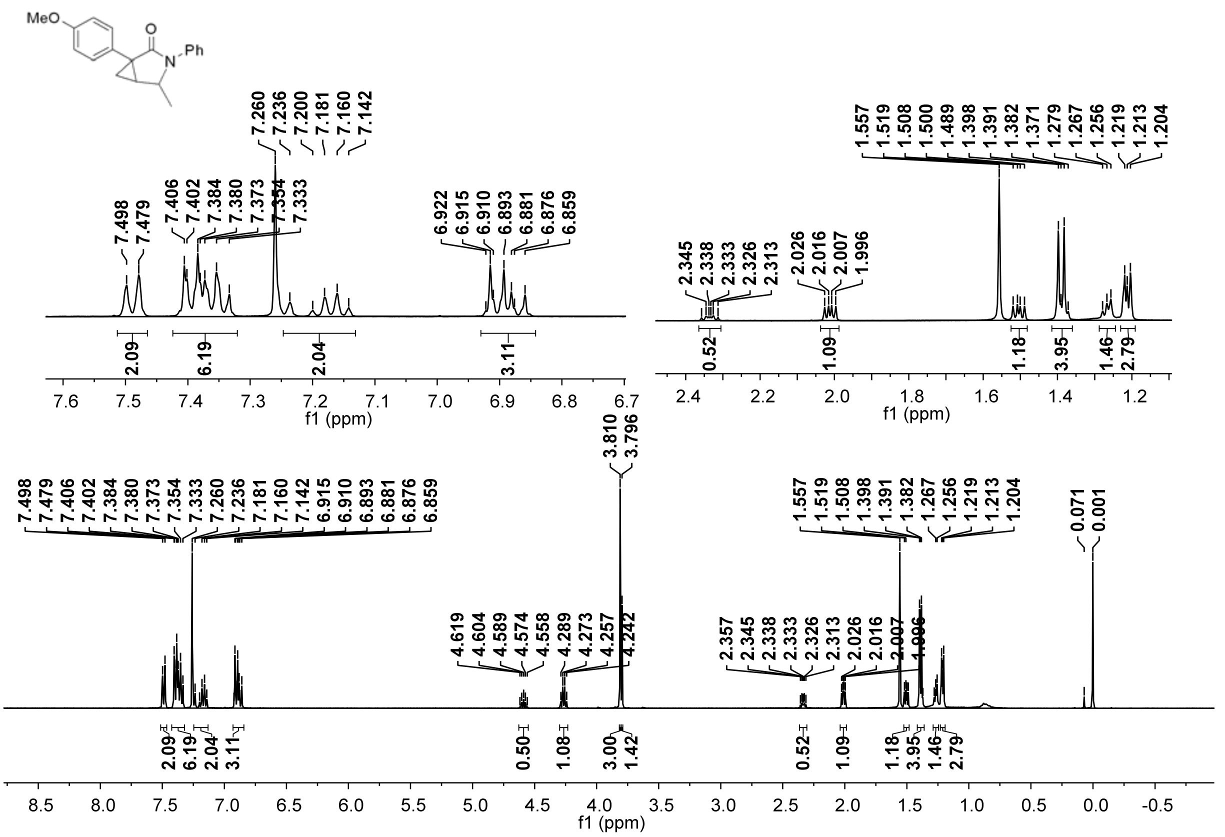

^{13}C NMR spectra of **2j** (150 MHz, $CDCl_3$)

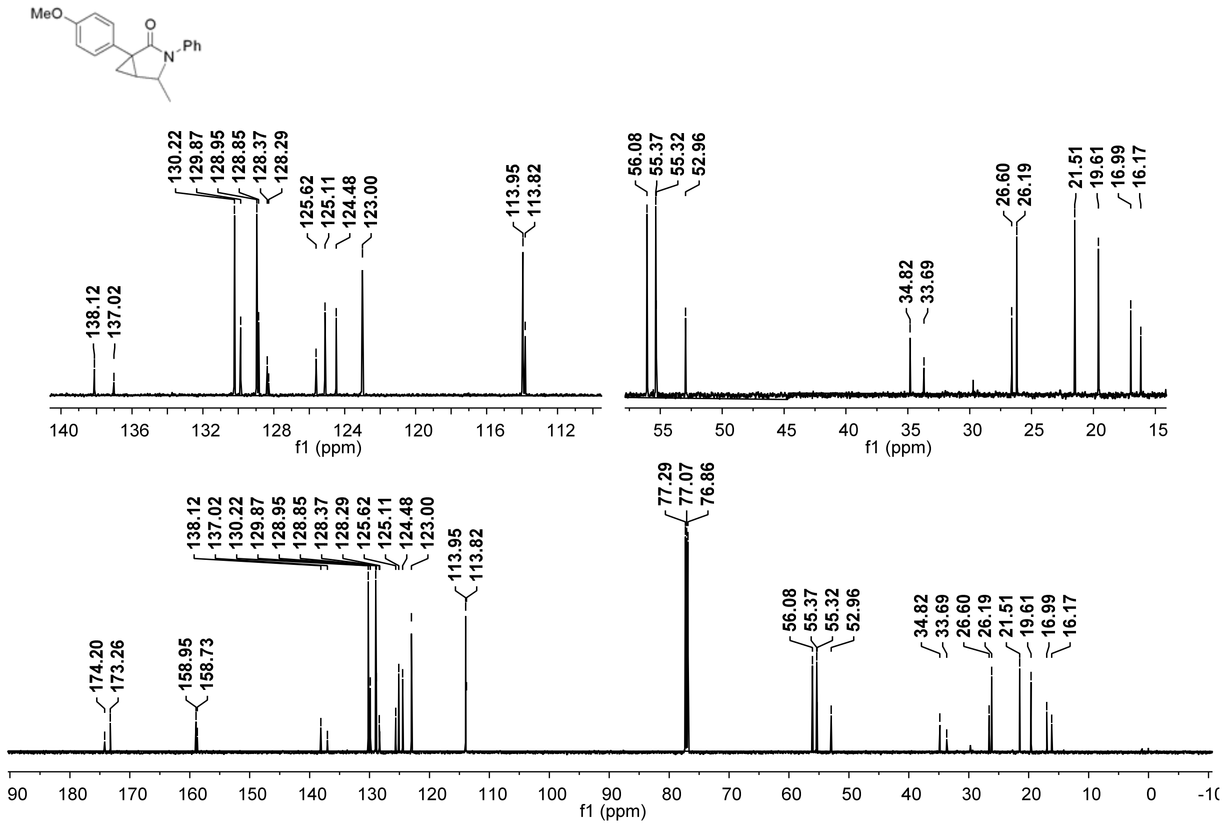

^{1}H NMR spectra of **2k** (600 MHz, $CDCl_3$)

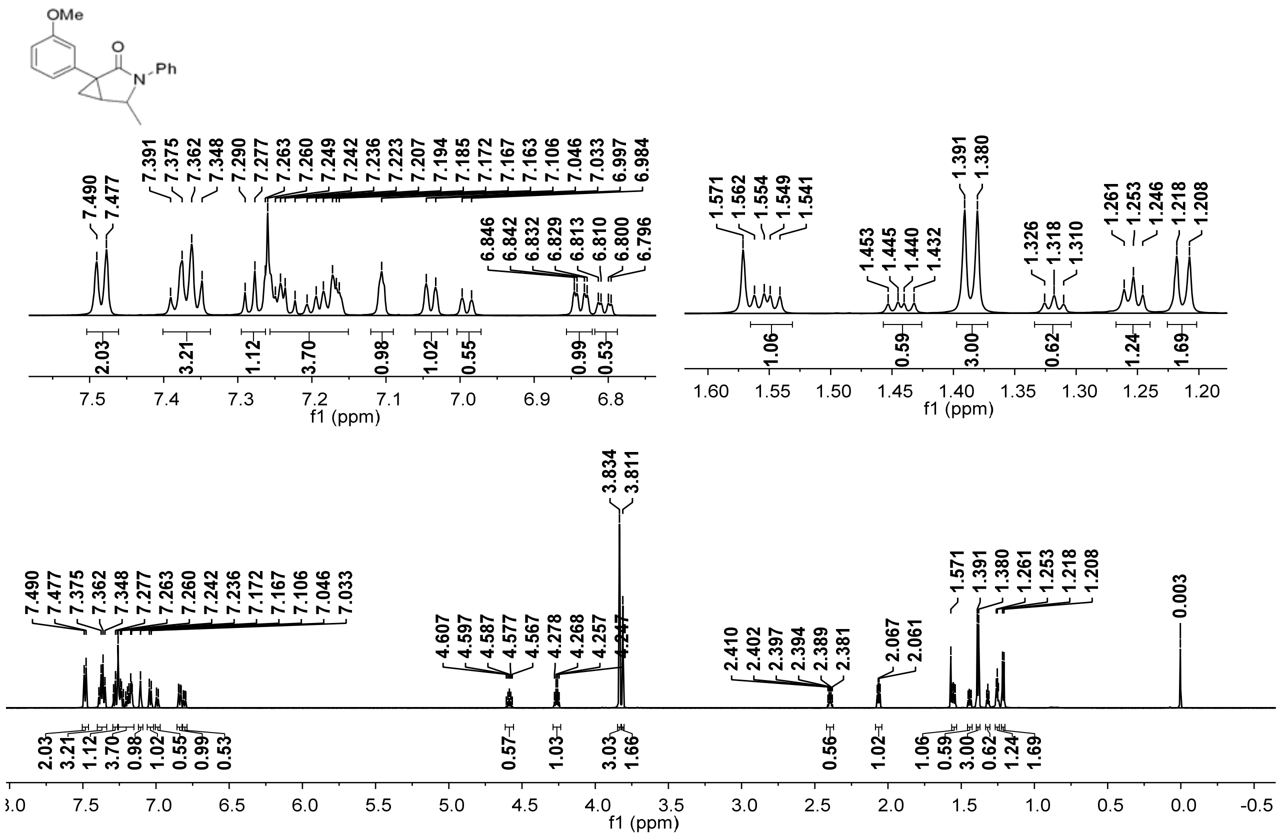

^{13}C NMR spectra of **2k** (100 MHz, $CDCl_3$)

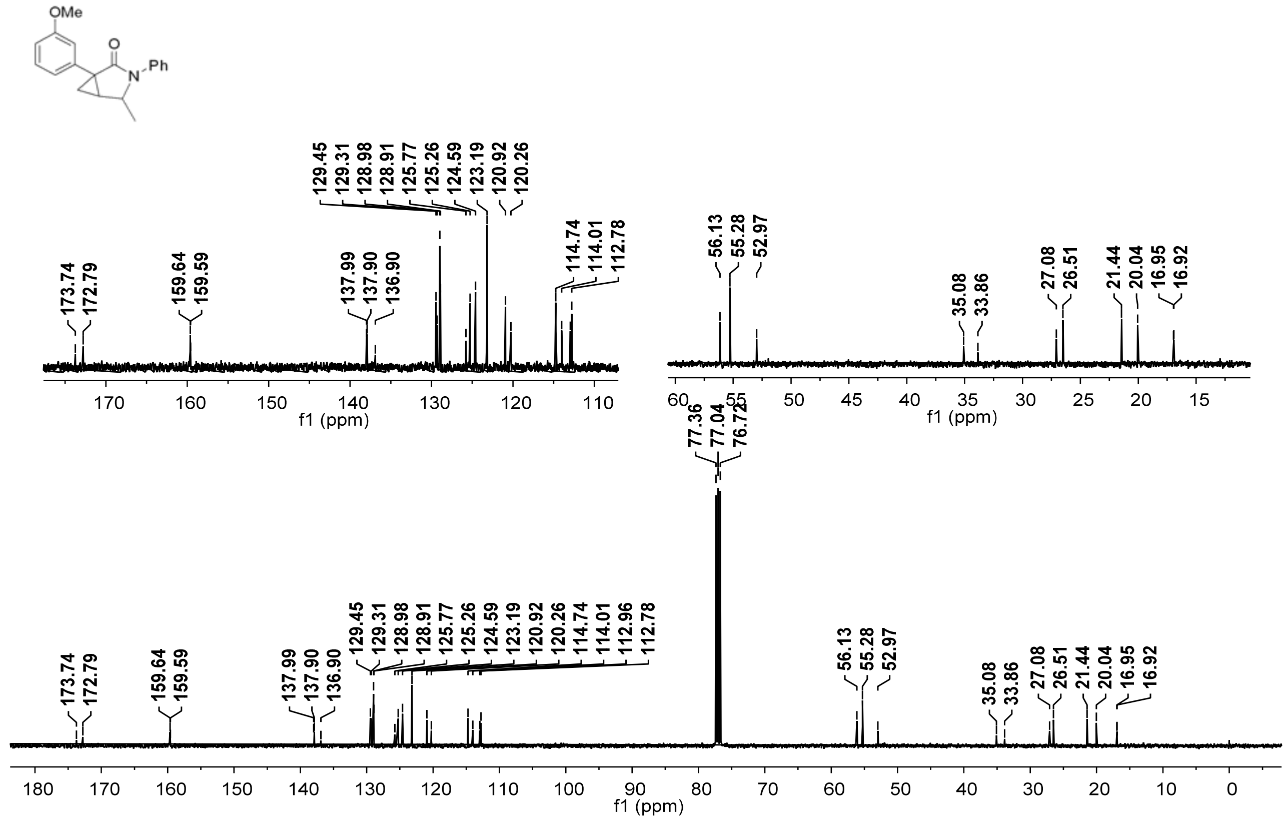

^{1}H NMR spectra of **2l** (600 MHz, $CDCl_3$)

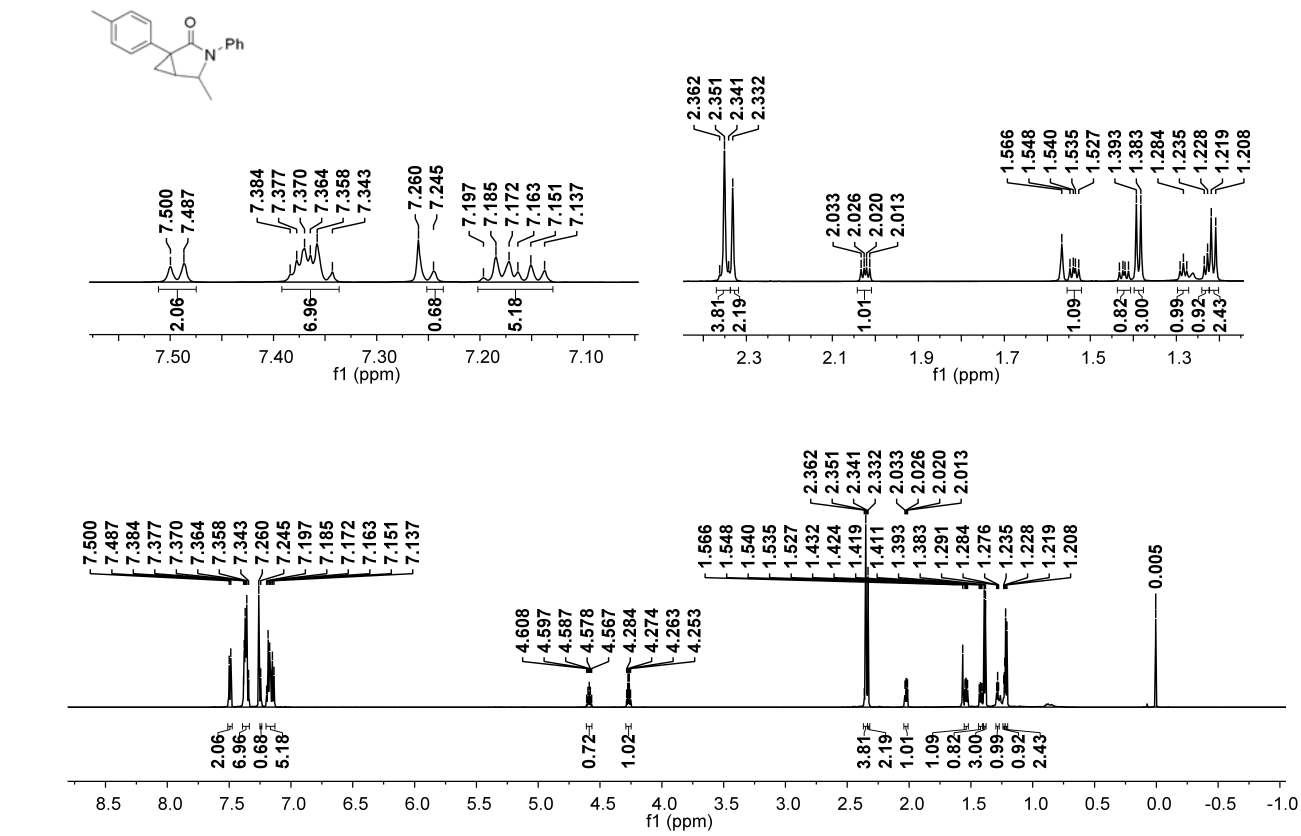

^{13}C NMR spectra of **2l** (150 MHz, $CDCl_3$)

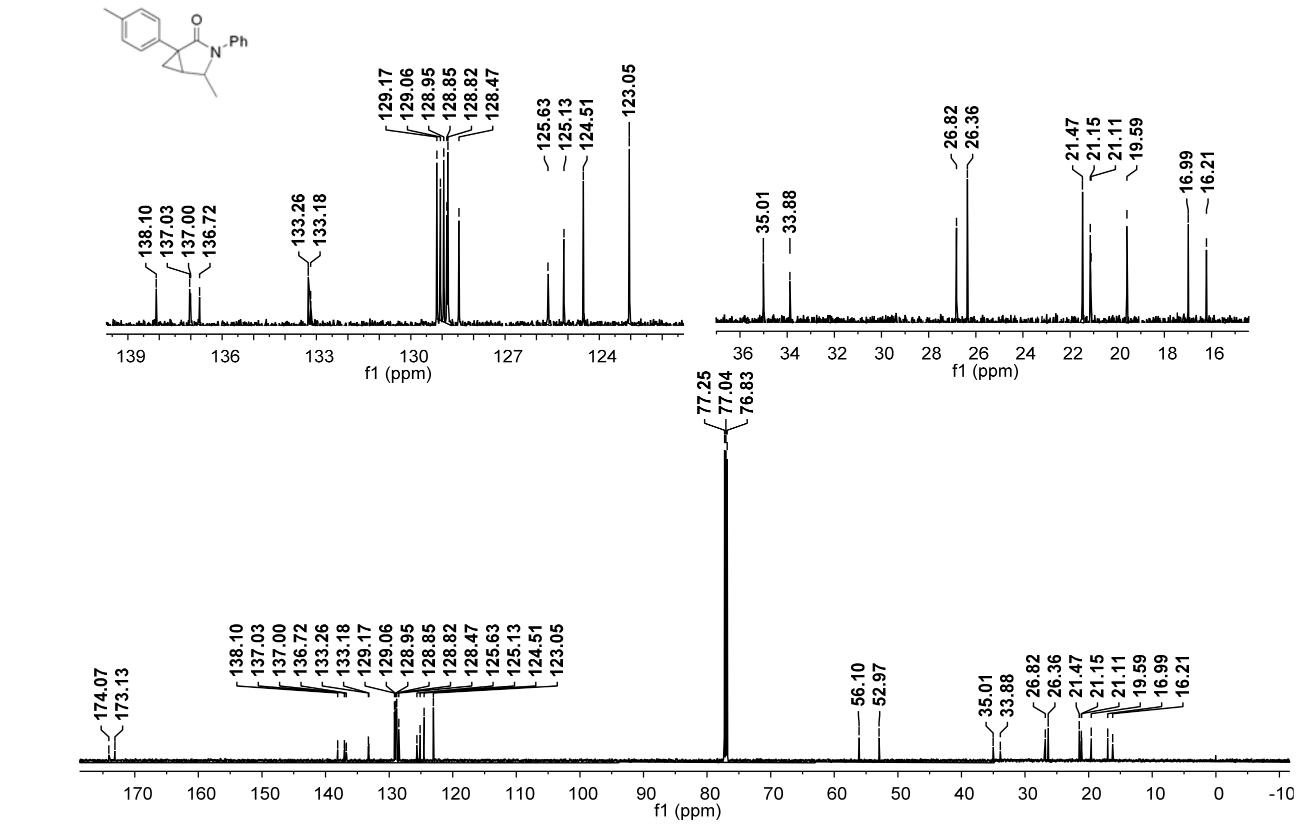

^{1}H NMR spectra of **2m** (400 MHz, $CDCl_3$)

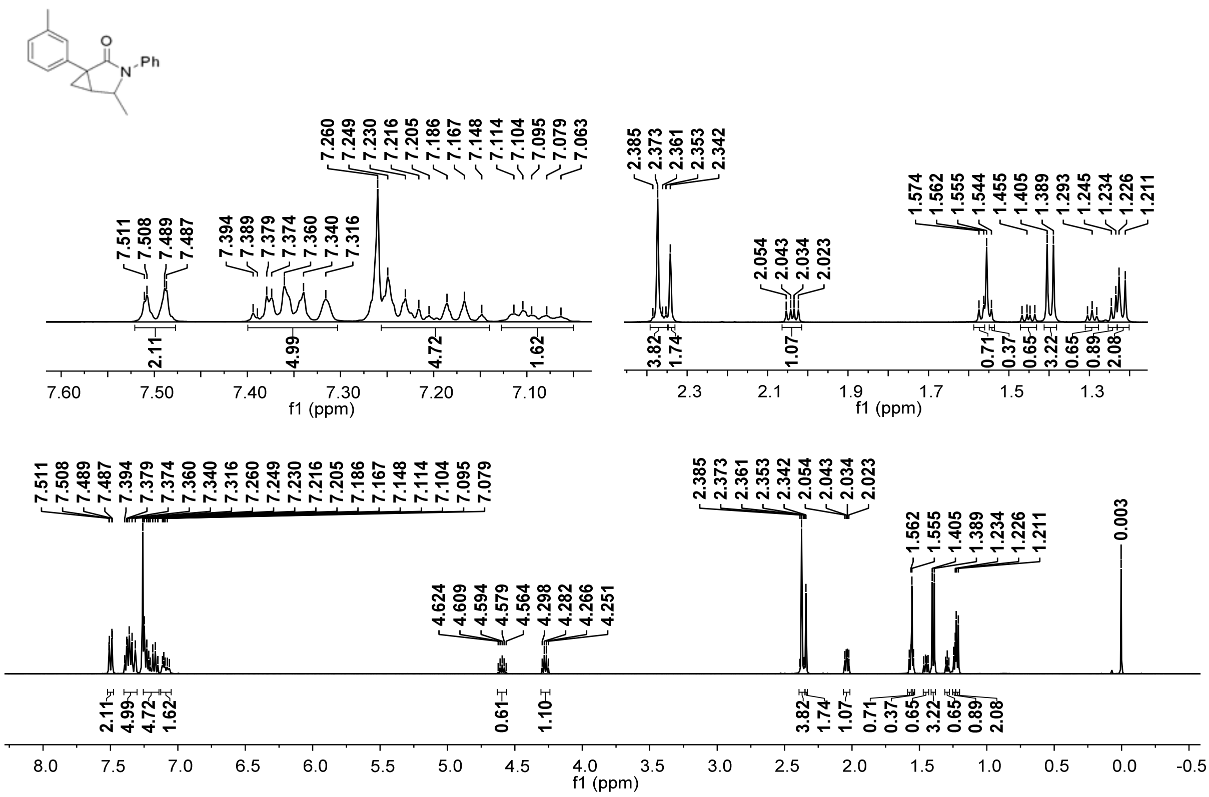

^{13}C NMR spectra of **2m** (100 MHz, $CDCl_3$)

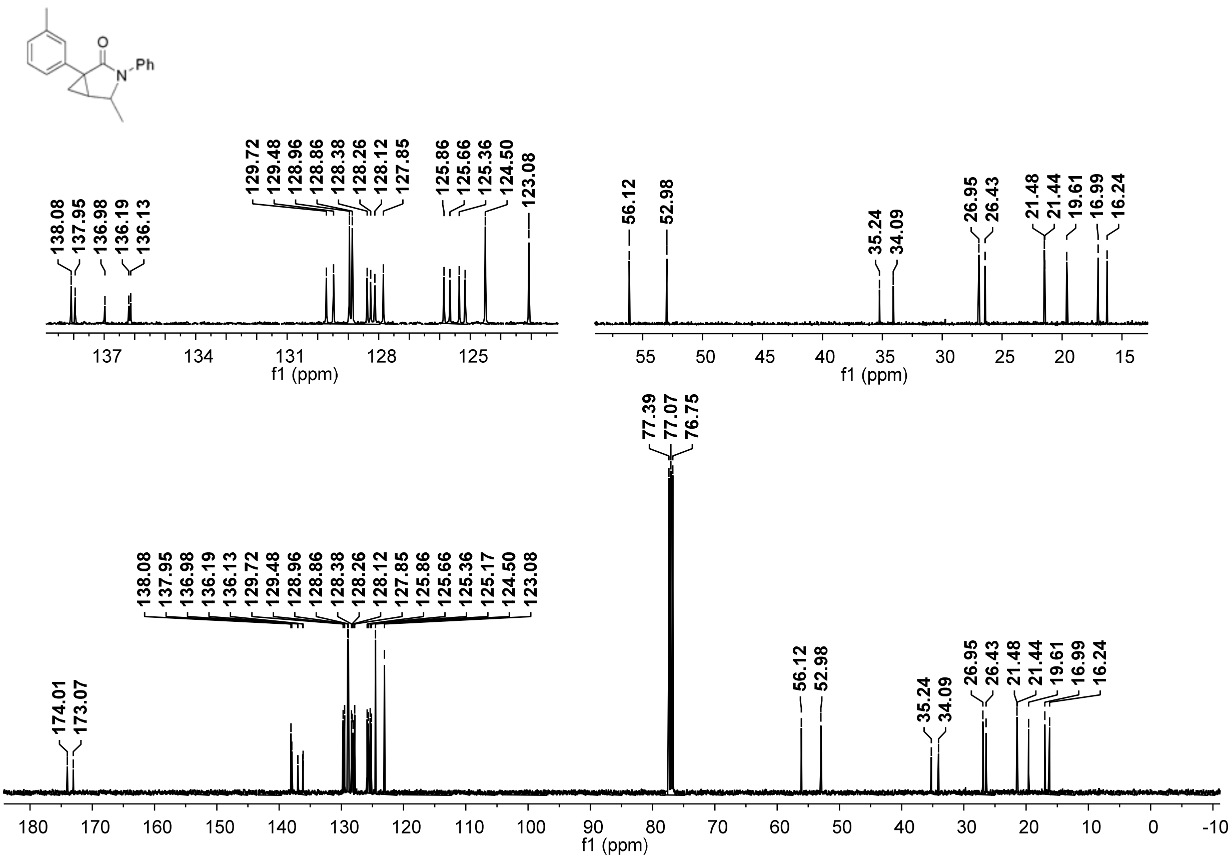

^{1}H NMR spectra of **2n** (600 MHz, $CDCl_3$)

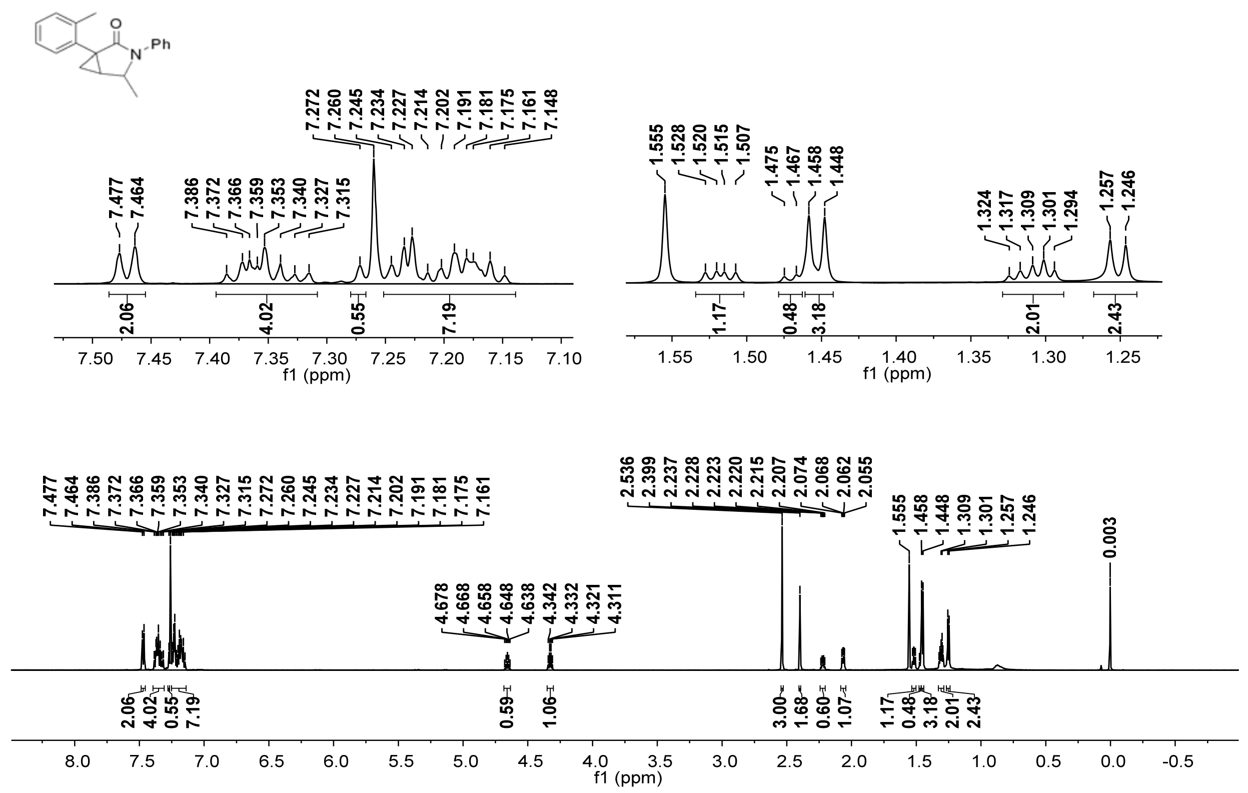

^{13}C NMR spectra of **2n** (150 MHz, $CDCl_3$)

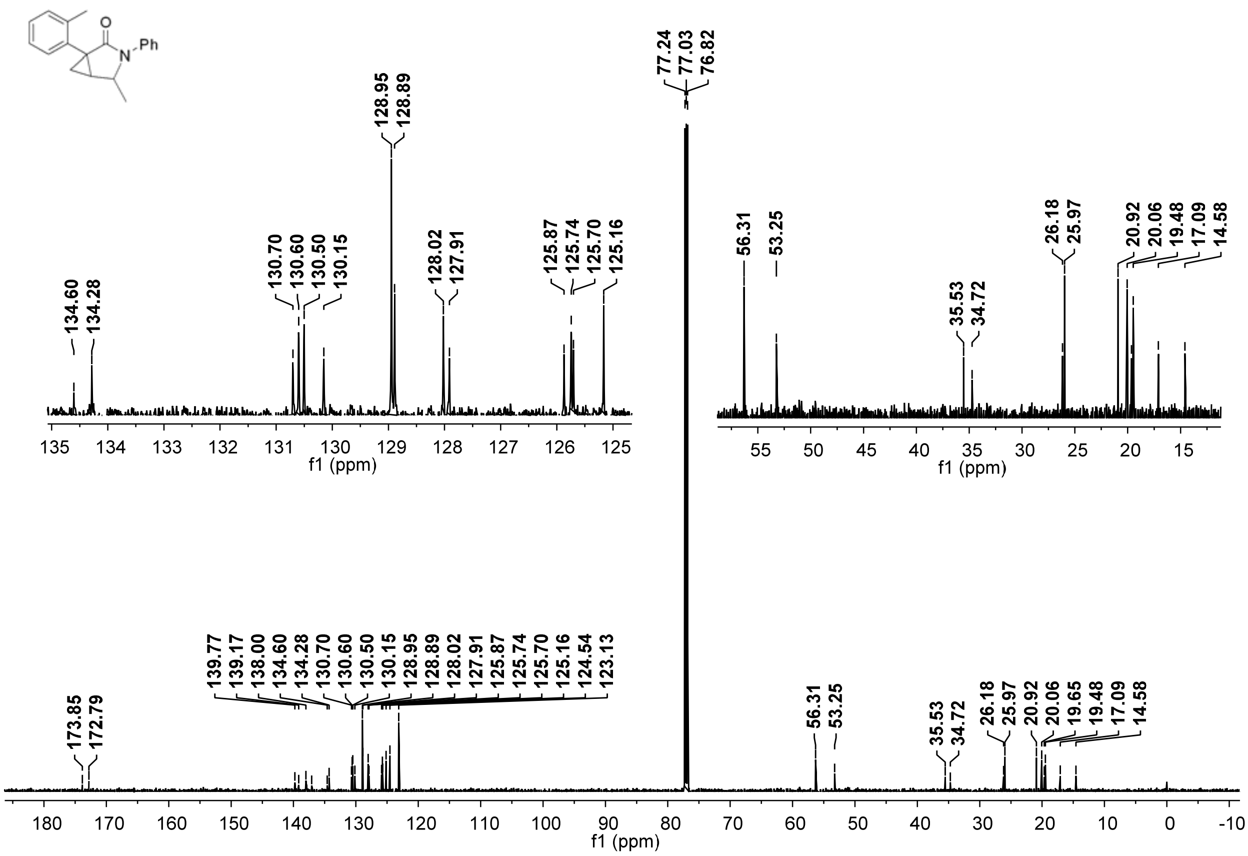

^{1}H NMR spectra of **2o** (400 MHz, $CDCl_3$)

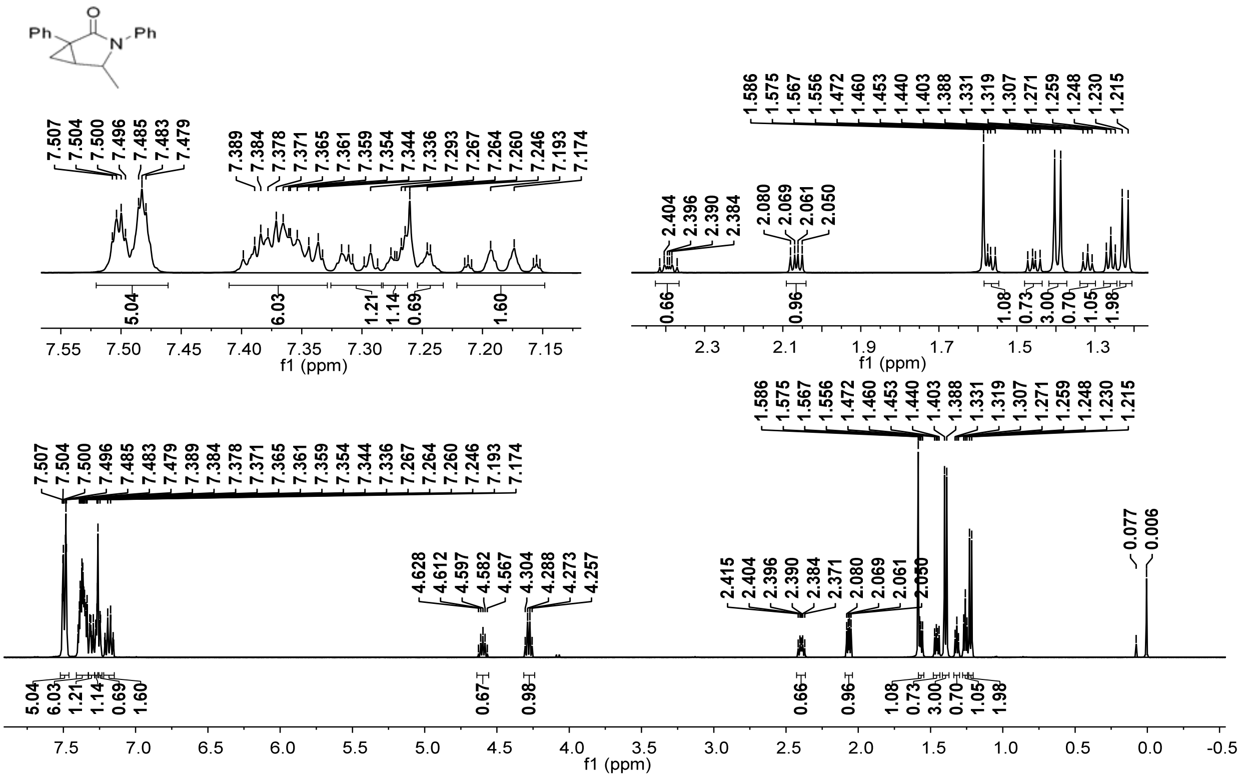

^{13}C NMR spectra of **2o** (150 MHz, $CDCl_3$)

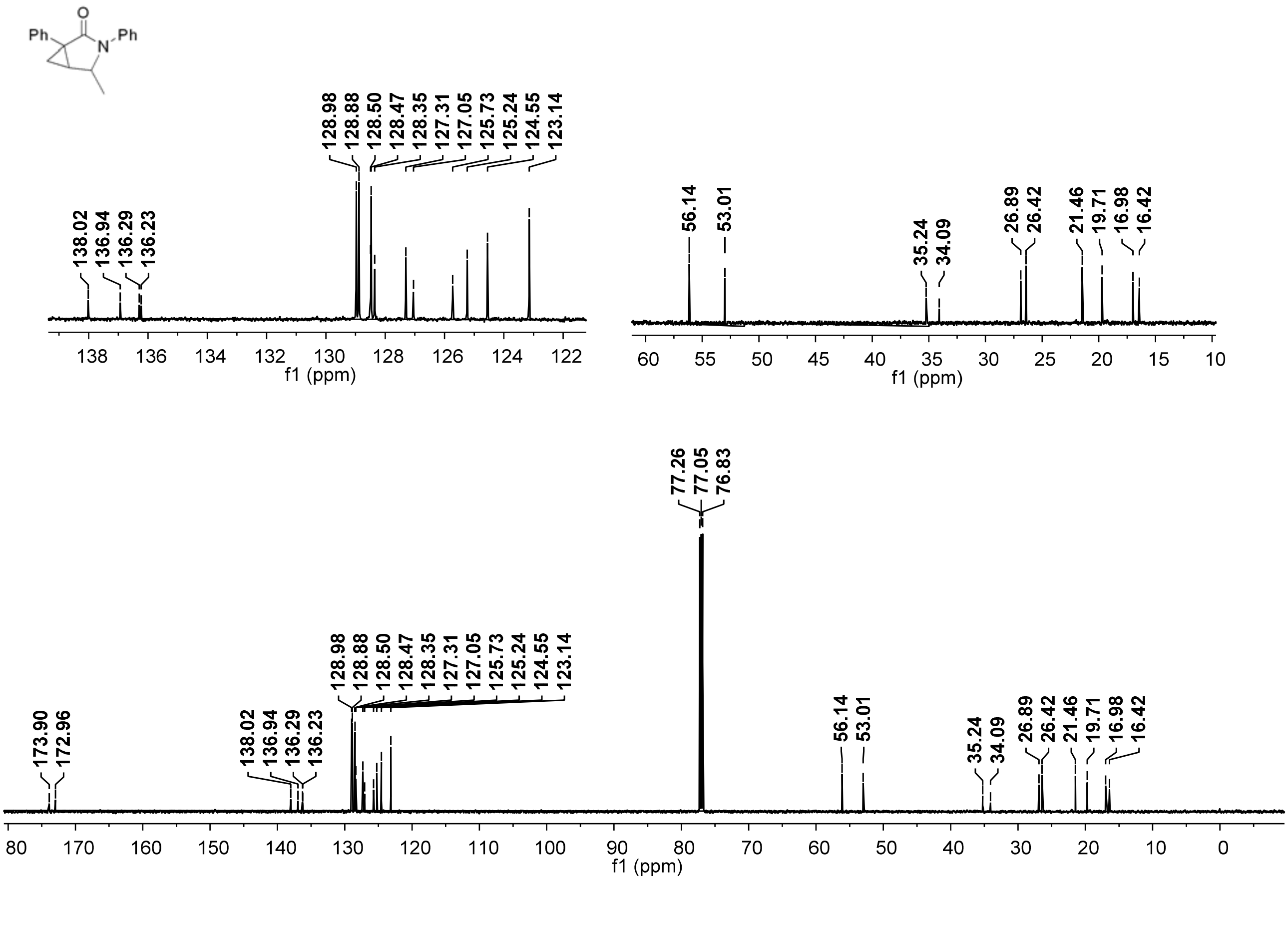

^{1}H NMR spectra of **2p** (600 MHz, $CDCl_3$)

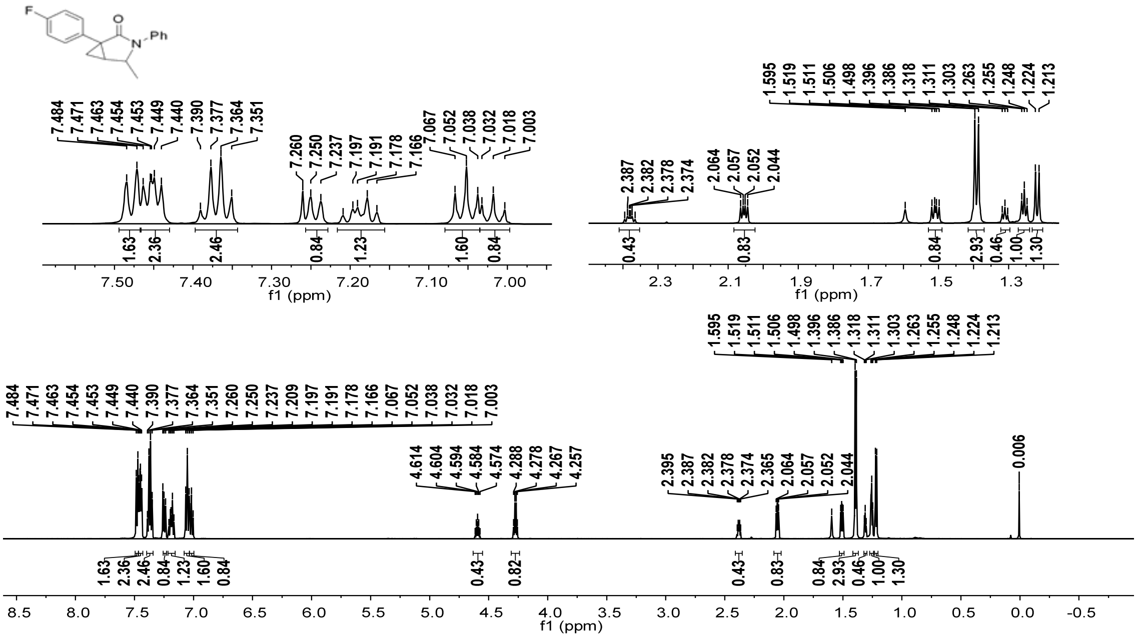

^{13}C NMR spectra of **2p** (100 MHz, $CDCl_3$)

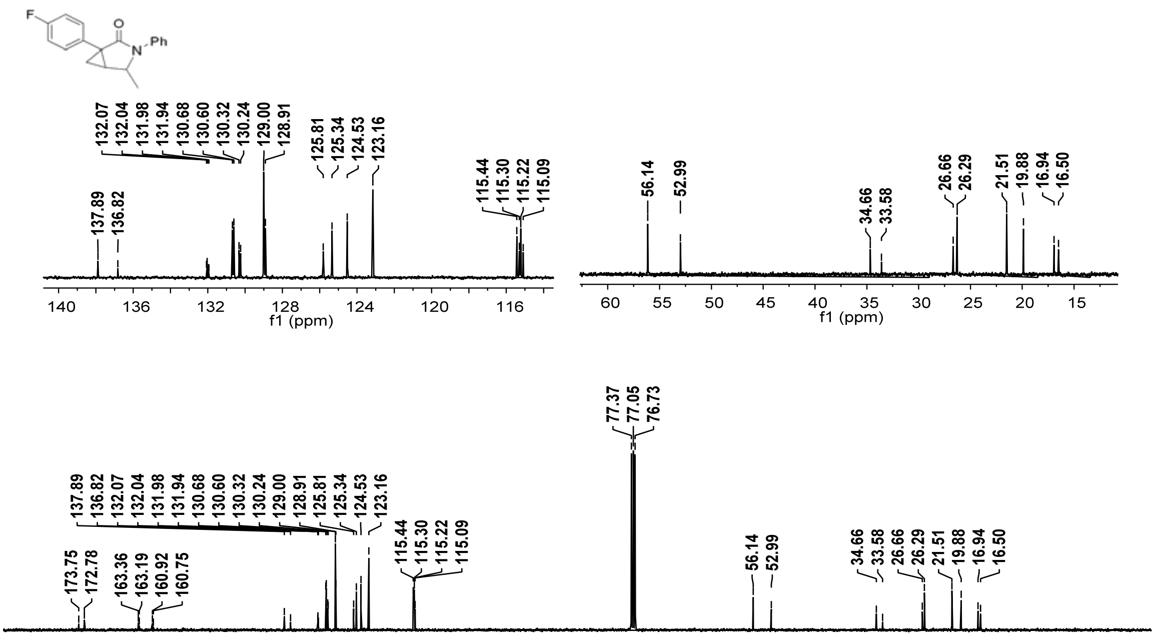

^{19}F NMR spectra of **2p** (376 MHz, $CDCl_3$)

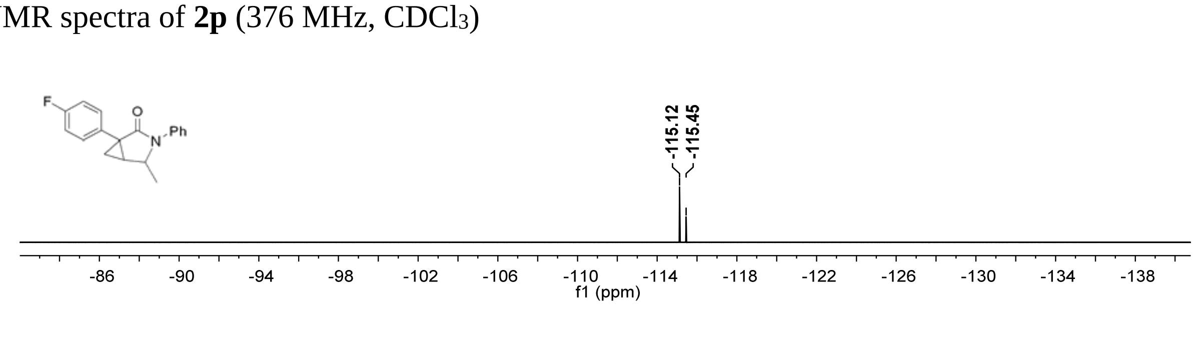

^{1}H NMR spectra of **2q** (600 MHz, $CDCl_3$)

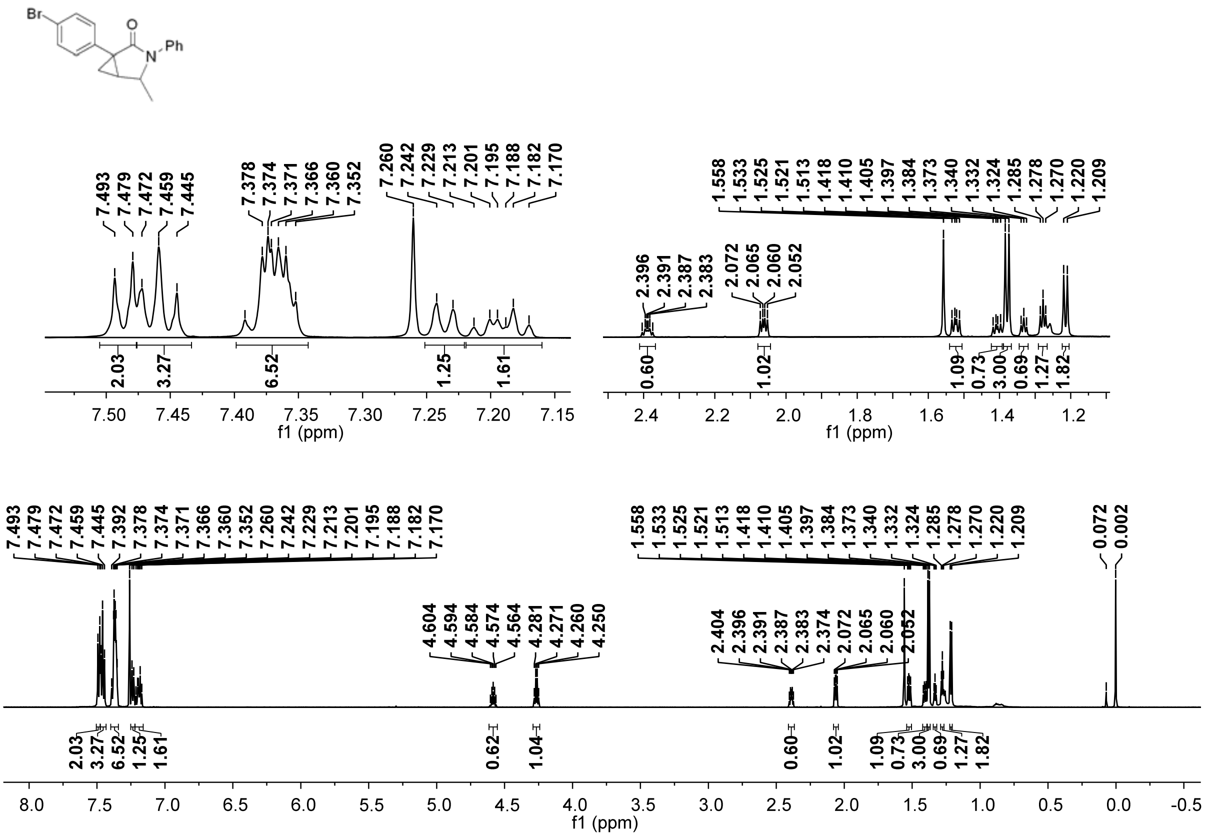

^{13}C NMR spectra of **2q** (100 MHz, $CDCl_3$)

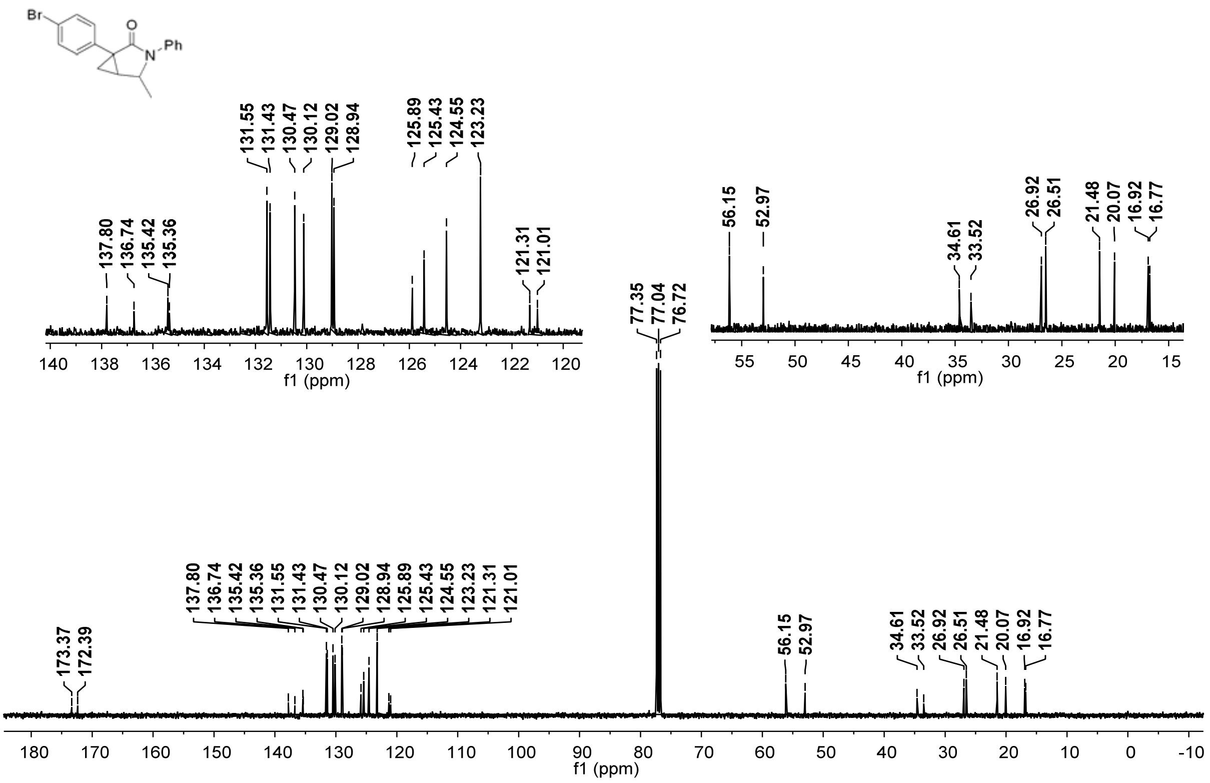

^{1}H NMR spectra of **2r** (600 MHz, $CDCl_3$)

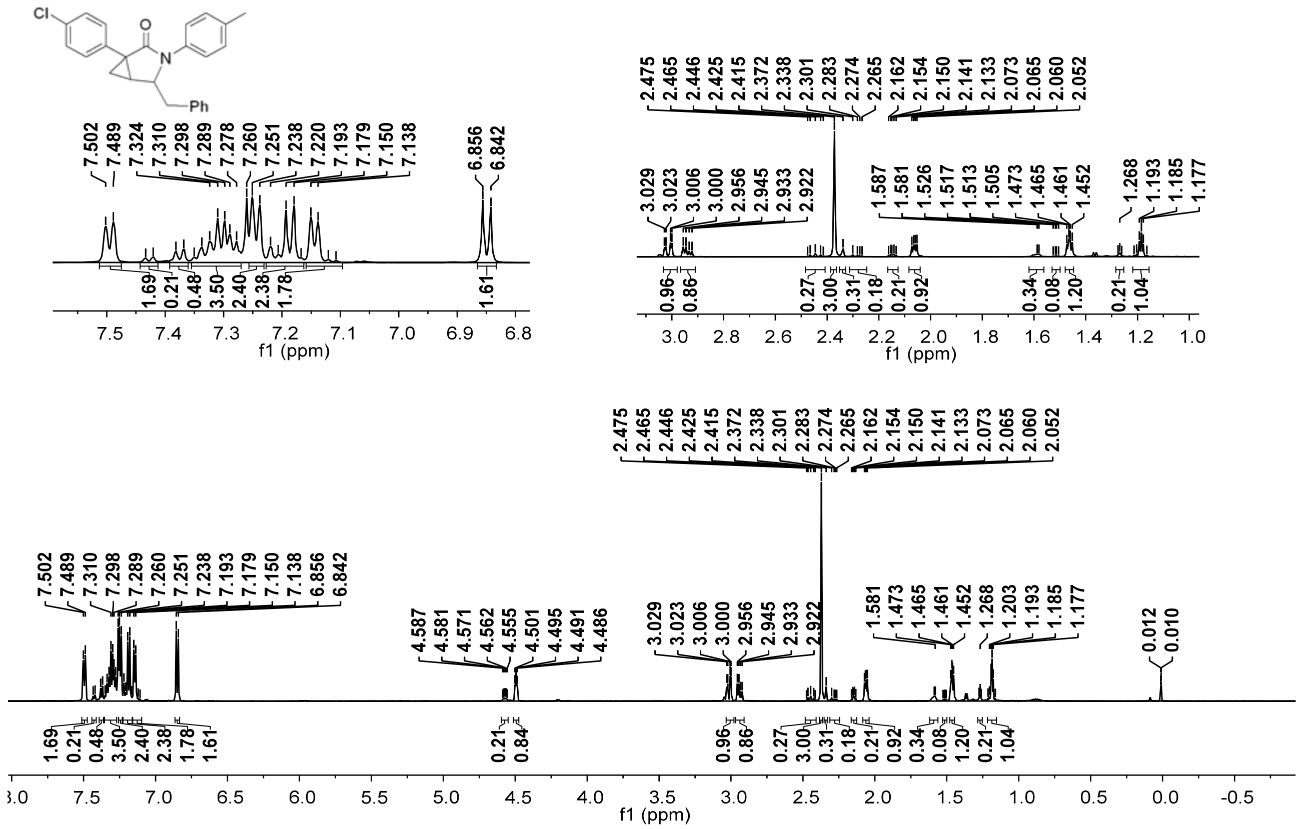

^{13}C NMR spectra of **2r** (150 MHz, $CDCl_3$)

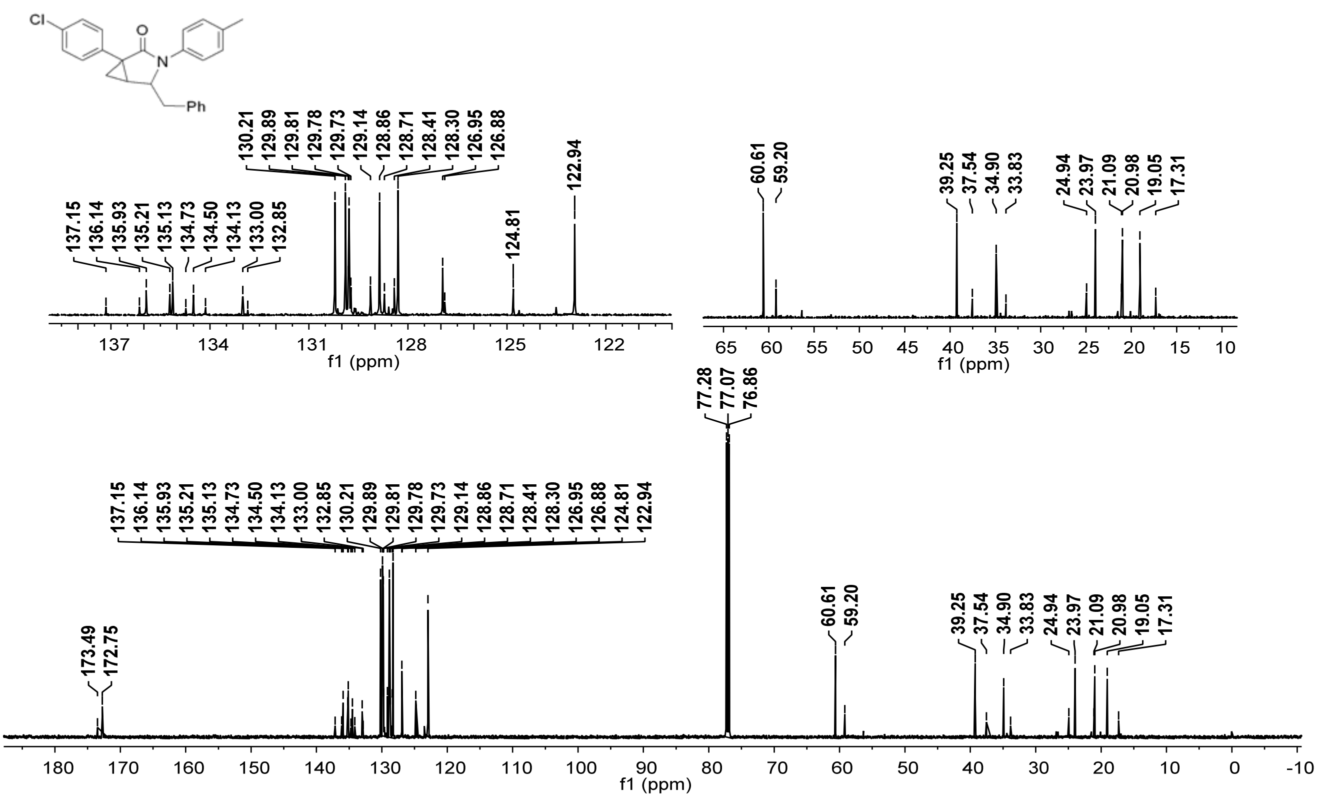

Supplement: Supplementary file 1 [file molecules-28-03691-s001.zip › molecules-2357704-supplementary.pdf]
